# EEG hyperexcitability and hyperconnectivity linked to GABAergic inhibitory interneuron loss following traumatic brain injury

**DOI:** 10.1093/braincomms/fcae385

**Published:** 2024-11-27

**Authors:** Hazel G May, Konstantinos Tsikonofilos, Cornelius K Donat, Magdalena Sastre, Andriy S Kozlov, David J Sharp, Michael Bruyns-Haylett

**Affiliations:** Department of Brain Sciences, Imperial College London, London W12 0NN, UK; Department of Bioengineering, Imperial College London, London SW7 2AZ, UK; Department of Neuroscience, Karolinska Institutet, Stockholm 171 65, Sweden; Department of Clinical Neuroscience, Karolinska Institutet, Stockholm 171 65, Sweden; Department of Brain Sciences, Imperial College London, London W12 0NN, UK; Department of Medicinal Radiochemistry, Institute of Radiopharmaceutical Cancer Research, Helmholtz-Zentrum Dresden-Rossendorf, 01328 Dresden, Germany; Department of Brain Sciences, Imperial College London, London W12 0NN, UK; Department of Bioengineering, Imperial College London, London SW7 2AZ, UK; Department of Brain Sciences, Imperial College London, London W12 0NN, UK; Department of Bioengineering, Imperial College London, London SW7 2AZ, UK; Department of Bioengineering, Institut Quimic de Sarria, Universitat Ramon Llul, Barcelona 08017, Spain; Department of Quantitative Methods, Institut Quimic de Sarria, Universitat Ramon Llul, Barcelona 08017, Spain

**Keywords:** EEG, hyperconnectivity, GABAergic, interneurons, TBI

## Abstract

Traumatic brain injury represents a significant global health burden and has the highest prevalence among neurological disorders. Even mild traumatic brain injury can induce subtle, long-lasting changes that increase the risk of future neurodegeneration. Importantly, this can be challenging to detect through conventional neurological assessment. This underscores the need for more sensitive diagnostic tools, such as electroencephalography, to uncover opportunities for therapeutic intervention. Progress in the field has been hindered by a lack of studies linking mechanistic insights at the microscopic level from animal models to the macroscale phenotypes observed in clinical imaging. Our study addresses this gap by investigating a rat model of mild blast traumatic brain injury using both immunohistochemical staining of inhibitory interneurons and translationally relevant electroencephalography recordings. Although we observed no pronounced effects immediately post-injury, chronic time points revealed broadband hyperexcitability and increased connectivity, accompanied by decreased density of inhibitory interneurons. This pattern suggests a disruption in the balance between excitation and inhibition, providing a crucial link between cellular mechanisms and clinical hallmarks of injury. Our findings have significant implications for the diagnosis, monitoring, and treatment of traumatic brain injury. The emergence of electroencephalography abnormalities at chronic time points, despite the absence of immediate effects, highlights the importance of long-term monitoring in traumatic brain injury patients. The observed decrease in inhibitory interneuron density offers a potential cellular mechanism underlying the electroencephalography changes and may represent a target for therapeutic intervention. This study demonstrates the value of combining cellular-level analysis with macroscale neurophysiological recordings in animal models to elucidate the pathophysiology of traumatic brain injury. Future research should focus on translating these findings to human studies and exploring potential therapeutic strategies targeting the excitation-inhibition imbalance in traumatic brain injury.

## Teaser

May *et al*. investigated mild blast TBI in rats using EEG and immunohistochemical staining. They found chronic broadband hyperexcitability, increased connectivity and decreased inhibitory interneurons, indicating interneuronal losses despite no immediate effects. This imbalance highlights the need for diagnostic tools and links cellular mechanisms to clinical injury hallmarks.

## Graphical Abstract

**Graphical Abstract:**
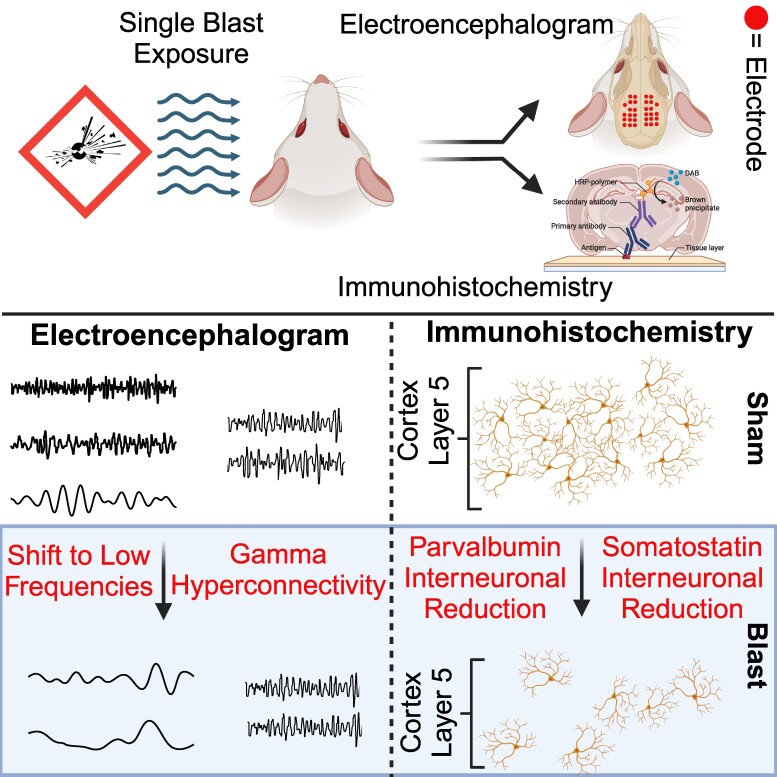

## Introduction

Recent publicity surrounding the development of neurodegenerative disease in military veterans and high-profile sports personalities has sparked a surge in traumatic brain injury (TBI) research.^1-8^ This has drawn attention to the potential long-term effects of TBI, specifically regarding the increased risk of developing conditions such as chronic traumatic encephalopathy, Alzheimer’s disease and other forms of dementia,^9,10^ with multiple TBI lifetime events also a key risk factor for future neurodegeneration.^4^

In the context of modern warfare, blast-induced traumatic brain injury (bTBI) is a common outcome for service personnel.^11,12^ An estimated 10–20% of soldiers deployed in Afghanistan and Iraq experienced TBI,^13-15^ and TBI was considered a signature injury of these conflicts^14^ with 77% of TBI cases of United states soldiers classified as mild TBI (mTBI).^12^ Part of this rise in incidence can be explained by the increased use of improvised explosive devices (IEDs), especially in asymmetric conflicts, such as Iraq and Afghanistan.^13,15,16^ In addition, the use of modern body armour and helmets along with advancements in battlefield medicine have resulted in increased survival rates for what were once previously fatal injuries.^13,17-20^

A big challenge in the management of TBI, especially mild TBI (concussion), is that symptoms often go unnoticed and unreported by sufferers.^15,21-23^ And even if reported, TBI can still escape typical clinical assessment measures.^24,25^ Indeed, bTBI is typically detected through a neurological assessment followed by brain imaging such as computed tomography or magnetic resonance imaging (MRI). However, this diagnostic protocol does not always detect milder forms of brain injury,^26^ as macroscopic changes spotted on structural scans in more severe forms of bTBI are rarely apparent in mild bTBI. Importantly, if no injury is identified then no recovery period will be mandated.^23,25^ This is concerning because repeated TBI events can result in more severe and long-term injury if a recovery period is not observed.^27^ These challenges highlight the urgency to find an effective method of identifying the initial occurrence of even a mild TBI, to manage injury and recovery effectively. Therefore, diagnosis of mild TBI calls for more sensitive techniques such as measuring changes in neural activity using EEG or MEG to provide a functional snapshot of brain-wide activity potentially sensitive enough to identify changes in the absence of traditional TBI imaging markers of pathology.

By using high temporal resolution functional imaging techniques such as Electroencephalography (EEG) and Magnetoencephalography (MEG), it is possible to identify changes in the absence of traditional TBI imaging markers of pathology. For example, an MEG approach^28,29^ outperformed a structural MRI diagnosis tool, where they demonstrated 87% of both blast and non-blast mTBI participants had the typical TBI hallmark feature of abnormally high delta (1–4 Hz) power relative to controls.^30-37^ This is in stark contrast to only 20% of the mTBI cohort showing any abnormalities in MRI. In addition, Franke *et al*.^34^ used EEG in bTBI and non-bTBI cohorts and reported increased delta and theta power changes as a lasting chronic effect of bTBI in the absence of clinically assessed symptoms. Power spectrum changes are not the only electrophysiological abnormality seen in TBI. Functional connectivity (FC) changes, quantified by the statistical dependency of signals from different brain regions,^38,39^ are also observed,^40,41^ resulting in the altered ability of brain-wide networks to form and transmit information. The finding of electrophysiological correlates of pathology in populations who show no clinical signs of injury is important, as those who have experienced one injury have a higher predisposition for repeated injury and therefore a potentially increased chance of developing future neurodegenerative disease.^40^

White matter damage has been proposed as one source of power and FC changes in EEG caused by TBI.^30,42^ However, this is not the only cellular change that can affect network dynamics, and there has been little investigation of the more subtle structural and functional changes to the delicate balance between the excitatory and inhibitory neurons integral to the brain’s homeostasis. Known as the excitation/inhibition balance (E/I), it plays an important role in normal neural function and an imbalance can result in neurological disorders and seizures.^43,44^ Inhibitory GABAergic interneurons play a crucial role in the E/I balance, and this extends to brain injury where two subtypes of inhibitory interneurons [expressing parvalbumin (PV) or somatostatin (SST)] have been found to be affected by TBI.^45-50^ PV expressing interneurons are the most abundant cortical interneurons (30–40% of interneurons)^51-53^ whereas SST expressing interneurons comprise ∼30% of interneuron (N) density.^52-54^ Given that it has been shown that perturbations to an interneuron population firing pattern or alteration in Ca^2+^-binding protein (e.g. PV) density can cause the E/I balance to be affected and result in behavioural as well as functional deficits,^43,48,55-58^ it is important to explore how different types of TBI affect the E/I balance. In addition, despite the prevalence of mild bTBI, it is unknown whether the E/I pathologies and possible impact on network connectivity match that reported in blunt impact TBI.^48,57^

To further examine the potential consequences of alterations in inhibitory function on brain activity, a preclinical model is necessary. In this research, we explored the potential value of EEG as a complementary addition to the existing suite of diagnostic tools within the context of mild bTBI. High-density EEG was used to measure the functional changes in brain activity, while immunohistochemistry for markers of white matter integrity, GABAergic interneurons and glial activation were employed to investigate the most likely related cellular changes. By combining the results from these techniques, we gain a more comprehensive understanding how alterations in the brain can affect cortical activity, leading to a better understanding of the underlying mechanisms driving mild TBI. Importantly, EEG is easy to use, portable, and cost-effective, making it a viable candidate for widespread use in the battlefield-adjacent and clinical settings. Using a bTBI rodent model, we present the first analysis of electrophysiological, neuronal, axonal and inflammatory pathology in mild bTBI over the acute and chronic phases of injury. While at the acute time point, we demonstrate minimal electrophysiological abnormalities, at chronic time points our findings reveal a characteristic electrophysiological power spectrum signature of TBI as well as an increase in gamma band FC. Additionally, we report the first evidence of accompanying changes in cortical regions and layer-specific GABAergic inhibitory interneuron (SST and PV) density.

## Materials and methods

### Animals

All animal experiments were conducted in compliance with the Home Office licence ([Scientific Procedures] Act 1986 and EU legislation). Male Sprague Dawley rats (∼330 g, Charles River, Margate, UK) were housed in individually ventilated cages of four until animals reached 350 g, and subsequently housed in pairs. The facility provided a 12-h light/dark cycle with temperature and humidity control. Animals were provided with standard rodent chow (Rat and Mouse No. 1 Maintenance, Special diets service, UK) and sterile water. All procedures and experiments were conducted during the light hours after a minimum 7-day acclimatisation period.

### Experimental design

**Figure 1:**
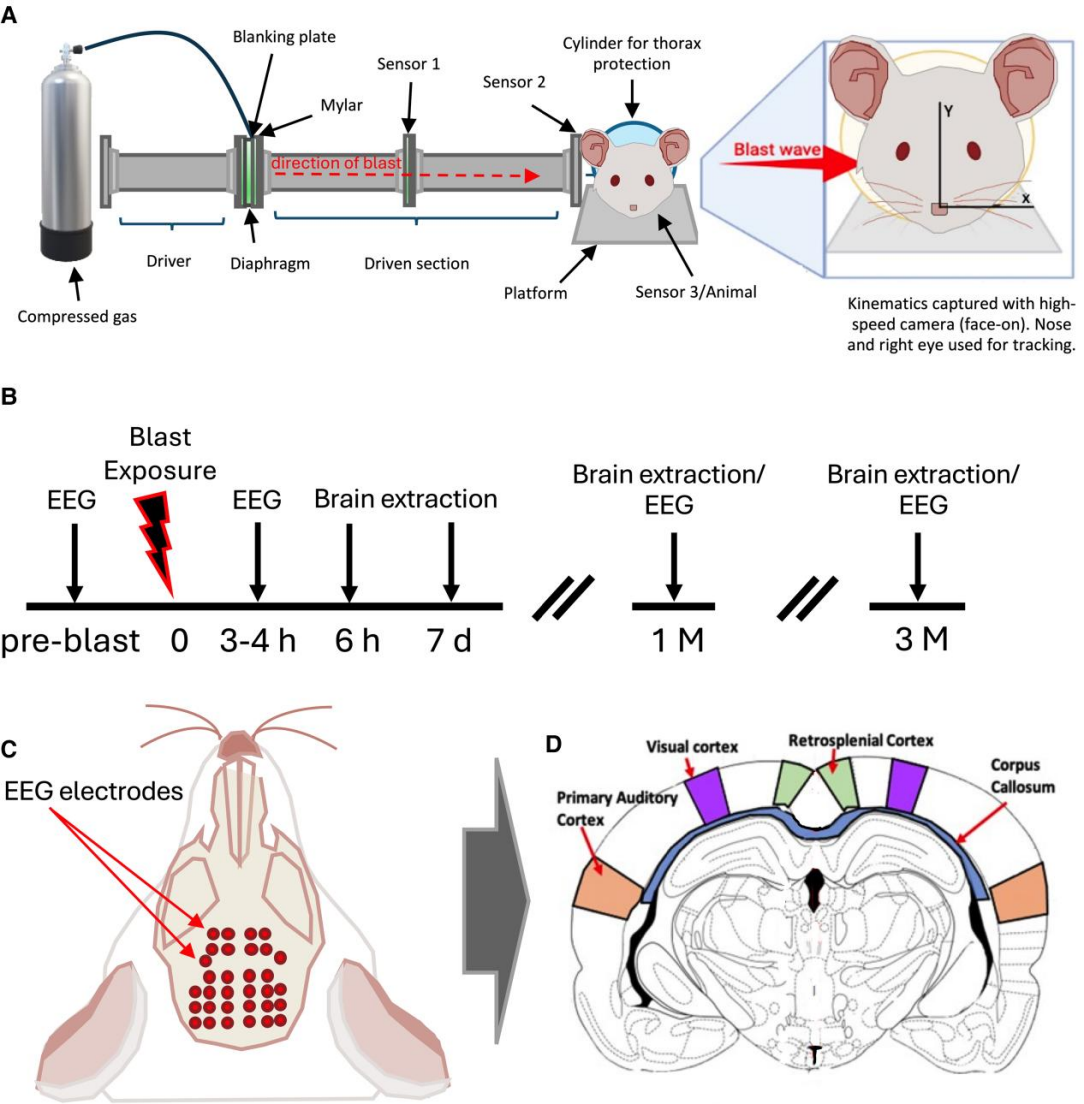
**Overview of methodology:** (**A**) a schematic of The Royal British Legion - Centre for Blast Injury Studies (TRBL-CBIS) shock tube used for blast exposures. It depicts the compressed gas cylinder that fills 7% of the driver tube using a blanking plate and 250 mm Mylar® diaphragms (which burst when threshold pressure is reached). Sensors 1, 2 and 3 are also visible down the driven shock tube. Sensor 2 is flush with the internal wall of the shock tube whereas Sensor 3 was placed during experiments with the animal not present. There is a visualized expansion to the right of the tube schematic, which indicates the direction of blast wave. The animal was placed perpendicular to the direction of the blast wave with the right side of the head closest to the shock tube exit. One fiducial point of reference used for tracing the kinematics, the nose, is shown. (**B**) Experimental timeline. (**C**) Schematic of the electrophysiological set up. Red dots represent the EEG electrodes. Red arrows point to the approximate location of the histological sections relative to the EEG array. (**D**) Representative schematic of sections stained. Coronal sections stained for the histological cohort were analysed in depicted ROIs, with the regions segmented and identified. ^59^

All blast experiments employed The Royal British Legion - Centre for Blast Injury Studies (TRBL-CBIS) shock tube at Imperial College London (Fig. 1). No study protocol was registered before the study. Animals were used to investigate the chronic effects of blast and were assigned to the following sub-groups prior to blast exposure to investigate either histopathological or electrophysiological markers of injury (Fig. 1).

The histopathological cohort (*n* = 39) was split into six groups (naïve: *n* = 3; sham-blast: *n* = 10; and blast exposure groups: 6-h post-blast: *n* = 6; 7-days post-blast: *n* = 6; 1-month post-blast: *n* = 8; 3-months post-blast: *n* = 6) and was subject to terminal anaesthesia, transcardial perfusion with PBS and 4% paraformaldehyde at different time points post-blast (either 6-h, 7-days, 1-month and 3-months post-blast).

The electrophysiological cohort (*n* = 50) was split into four groups (1-month post-sham-blast: *n* = 8; 3-months post-sham-blast: *n* = 9; 1-month post-blast: *n* = 13; 3-months post-blast: *n* = 20) and was subjected to terminal anaesthesia, EEG implantation and recording and euthanasia at the different time points following a sham-blast or blast procedure.

In addition, a subset of animals (*n* = 12) was used to investigate the acute electrophysiological effects of blast within 4 h after injury, and underwent recordings prior to and after blast exposure in a within-subjects design under continuous terminal anaesthesia.

The experimental unit was a single animal. Sample sizes were chosen to be aligned to or larger than those found in the literature. Further details about the shock tube as well as the blast procedure can be found in the Supplementary material.

### Blast exposure

Sprague–Dawley rats were subjected to a mild lateral blast under isoflurane using The TRBL-CBIS shock tube. The animals had their thorax protected during the blast and their heads were not restricted (Supplementary Fig. S1). Each animal was allowed to recover in an oxygen enriched warmed cage, given analgesia for 72 h post-injury and monitored for adverse effects until the end of experiments. Full details of the blast procedure can be found in the Supplementary material.

### Electrophysiological recordings

#### Surgery

All animals underwent electrophysiological recordings under terminal anaesthesia (urethane, intraperitoneal injection, 2.7 ml/kg, 1.35 g/kg, Sigma-Aldrich).

Once a surgical plane of anaesthesia had been achieved, animals were placed on a stereotaxic apparatus (Kopf Instruments, USA) for surgery. A midline incision was made using a scalpel, followed by clearing of connective tissue via blunt dissection to clear the dorsal surface of the skull. Further details about the preoperative preparations and surgery are included in the Supplementary material.

#### Recording setup

Following surgery (∼1 h), animals were head-fixed to a magnetic arm via a head plate affixed to the skull (caudal of the lambdoid suture) with dental cement and placed inside a shielded soundproofed recording chamber. Thirty-two-channel EEG multi-electrode arrays (Rat EEG Functional, Neuronexus) were implanted on the dorsal surface of the skull using a saline drop for adhesion, with a reference electrode placed in the neck. The array covered an area from the lambdoid suture to rostral of the coronal suture between the lateral ridges (Fig. 1C). Signals were acquired using Neuronexus headstages, Smartbox^TM^ acquisition board and software (for details please see Supplementary material; for traces of raw data see Supplementary Fig. S6). Spontaneous activity was recorded for multiple 5-min intervals (at least 2 h after surgery) over an ∼6-h-long experimental session including sensory stimulation paradigms (data not shown here), a duration sufficient for reliable spectral power and connectivity estimates.^60,61^ Power estimates were stable along the length of the recording sessions (Supplementary Fig. S8).

### EEG data analysis

#### EEG pre-processing

Data were pre-processed using functions from the EEGLAB toolbox.^62^ Signals were first resampled at 1 kHz and then line noise at 50 Hz and harmonics were suppressed using the CleanLine method version from the PREP toolbox (function *cleanLineNoise*,^63^). Data were then scanned for artefacts using the artefact subspace reconstruction algorithm (function *clean_artifacts*^64^). Segments containing artefacts were discarded from subsequent analysis. Finally, data were decomposed into independent components, using the infomax algorithm (function *pop_runica*^65^) with default settings. Resulting components were conservatively rejected for physiological artefacts based on their activation time series and spectra. Further details on the choice of parameters and pre-processing steps are available in the Supplementary material.

#### EEG analysis of power

All datasets were ensured to be of equal duration to achieve comparable signal to noise ratios. Power spectral densities were computed for each channel from 1 to 200 Hz using the fast Fourier transform and the Welsch’s method on 2 s consecutive windows with 50% overlap (function *pop_spectopo*). The resulting spectra were both directly analysed to obtain estimates of absolute power, and also normalized to the total power. Both absolute and normalized power was then obtained for canonical EEG frequency bands (delta: 1–4 Hz, theta: 4–8 Hz, alpha: 8–12 Hz, beta: 12–25 Hz, gamma: 25–80 Hz, high-frequency oscillations (HFO): 80–200 Hz) by taking the mean of the values within each band. To obtain global power, spectra were averaged across the electrode array. We further explored changes in power at individual electrodes for cohorts where a difference in global power was present. For the analysis of power at the electrode level, channels rejected during pre-processing (fewer than three channels for any animal) were interpolated using spherical interpolation (function *pop_interp* with default settings; results were identical when an inverse distance method or no interpolation at all (Supplementary Fig. S7) was used). When multiple runs were available per animal, a summary measure of all runs was obtained by averaging over runs.

#### EEG analysis of connectivity

Data were epoched into 3 s segments, and the cross-spectral density for each electrode pair computed using Welch’s method with a 1.5 s sliding window with 50% overlap (MATLAB function *cpsd*). These values were used as input to the low-level FieldTrip^66^ function *ft_connectivity_wpli*, to compute the debiased, weighted phase lag index (dwPLI),^66,67^ between electrode pairs, a phase synchronisation measure of connectivity robust to volume conduction. Connectivity values were averaged within the frequency bands defined above to assess band-specific differences between groups. To obtain global connectivity, dwPLI values were averaged across all pairs of the electrode array. When multiple runs were available per animal, a summary measure of all runs was obtained by averaging over runs.

#### Immunostaining

Following transcardial perfusion with PBS and 4% paraformaldehyde, brains were post-fixed (24 h), blocked, processed and paraffin-embedded. From paraffinized blocks, sections were cut (7 µm) coronally. To investigate neuronal density, sections were stained with an antibody against NeuN (Millipore), and for specific GABAergic inhibitory interneuron density with PV (Swant) and SST (Millipore). To assess changes in the density of glial cells in response to the blast, we stained for glial fibrillary acidic protein (GFAP) for astrocytes, and ionized calcium-binding adaptor molecule 1 (BA1) as a marker for microglia, and visualized glial activation using 3,3′-diaminobenzidine (Vectorlabs). Immunofluorescent staining was conducted with neurofilament light (NFL) to assess axonal damage in the white matter. Details for the immunostaining are found in the Supplementary material.

#### Immunostaining imaging

Full coronal slices were used for light-microscopy (NeuN, PV, SST, IBA1 and GFAP), and imaged at 20× with a slide scanner (Aperio AT2 20x/0.75 NA Plan Apo, Leica Biosystems, Germany). For the immunofluorescent images, half sections including the whole corpus callosum (CC) and neocortex were acquired using the tile function on the LSM 780-inverted confocal laser scanning microscope (Carl Zeiss), and Zen software at 10×.

#### Immunostaining image analysis

Three slices were used per stain/animal and averaged. The regions of interest (ROI) analysed were the primary auditory cortex (Au1), primary visual cortex (V1), retrosplenial cortex (RSC) and the CC (Fig. 1D). The densities of NeuN, PV and SST positive neurons were analysed separately for each cortical layer (layer 2/3–6) for each ROI. PV and SST analysis densities were manually counted using HALO. NeuN positive cells were quantified using HALO’s cytonuclear algorithm. For glial analysis no cortical layers were defined for the Au1, V1 or RSC, and the whole CC was outlined. Astrocytes (GFAP) and microglia (BA1) percentage area stained were assessed using HALO’s percentage area-stained algorithm. Microglial (BA1) densities were calculated using HALO’s microglial algorithm. NFL in the CC was measured using HALO’s immunofluorescent intensity algorithm and normalized to the periaqueductal grey. These areas of grey matter with minimal fluorescence served as the internal control. For the statistical analysis, all groups were compared to the Naïve/Sham group. Further details on immunostaining image analysis are described in the Supplementary material.

### Statistical analysis

Statistical analysis was performed using Matlab^TM^ and GraphPad Prism (9.4.1). Electrophysiological changes were assessed using paired *t*-tests for the acute cohort and 2-way ANOVAs (injury group × post-injury time point with interaction term) for the chronic cohort. Two sample *t*-tests were performed for *post hoc* pairwise comparisons in the case of an interaction. Histological changes were assessed first using 2-way ANOVAs (time post-injury × hemisphere with interaction term). In all cases, the factor ‘hemisphere’ was non-significant and therefore one-way ANOVAs were conducted with time post-injury as the factor, followed by *post hoc* pairwise comparisons against the control group. Data were presented as ±SEM unless stated otherwise, and the threshold for significance was set at *P* = 0.05. Further details on statistical analyses are provided in the Supplementary material.

## Results

### Minimal electrophysiological changes in the acute post-blast period

**Figure 2:**
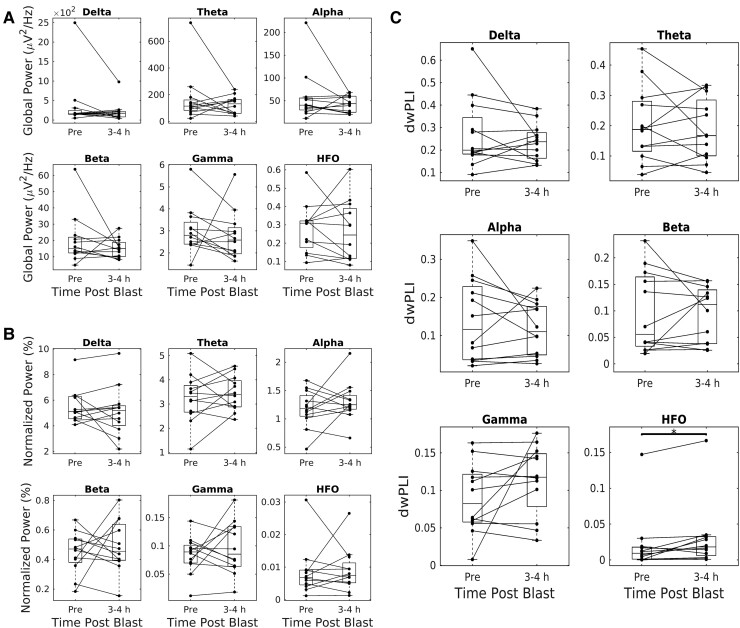
**Electrophysiological power and connectivity at the acute post-injury time point.** (**A**) Global absolute power across all frequency bands. No significant differences were found for any band. (**B**) Global normalized power across all frequency bands. No significant differences were found for any band. (**C**) Global connectivity across all frequency bands. A significant difference was found for the HFO band. All panels: delta: 1–4 Hz, theta: 4–8 Hz, alpha: 8–12 Hz, beta: 12–25 Hz, gamma: 25–80 Hz and HFO: 80–200 Hz. Permutation-based paired *t*-test (pre-blast versus 3–4 h post-blast, 10 000 iterations) with Bonferroni correction for multiple comparisons (*n* = 6 bands) (*n* = 12 animals).

Analysis of ‘absolute’ (Fig. 2A) and ‘normalized’ (Fig. 2B) global EEG power revealed no differences in resting-state EEG power in any of the bands examined. Analysis of global FC (Fig. 2C) revealed significantly elevated values at 3–4 h post-blast compared to baseline (*P* < 0.05; paired *t*-test—11 degrees of freedom) for the high-HFO band.

### EEG power spectrum increases at chronic time points due to mild bTBI

**Figure 3:**
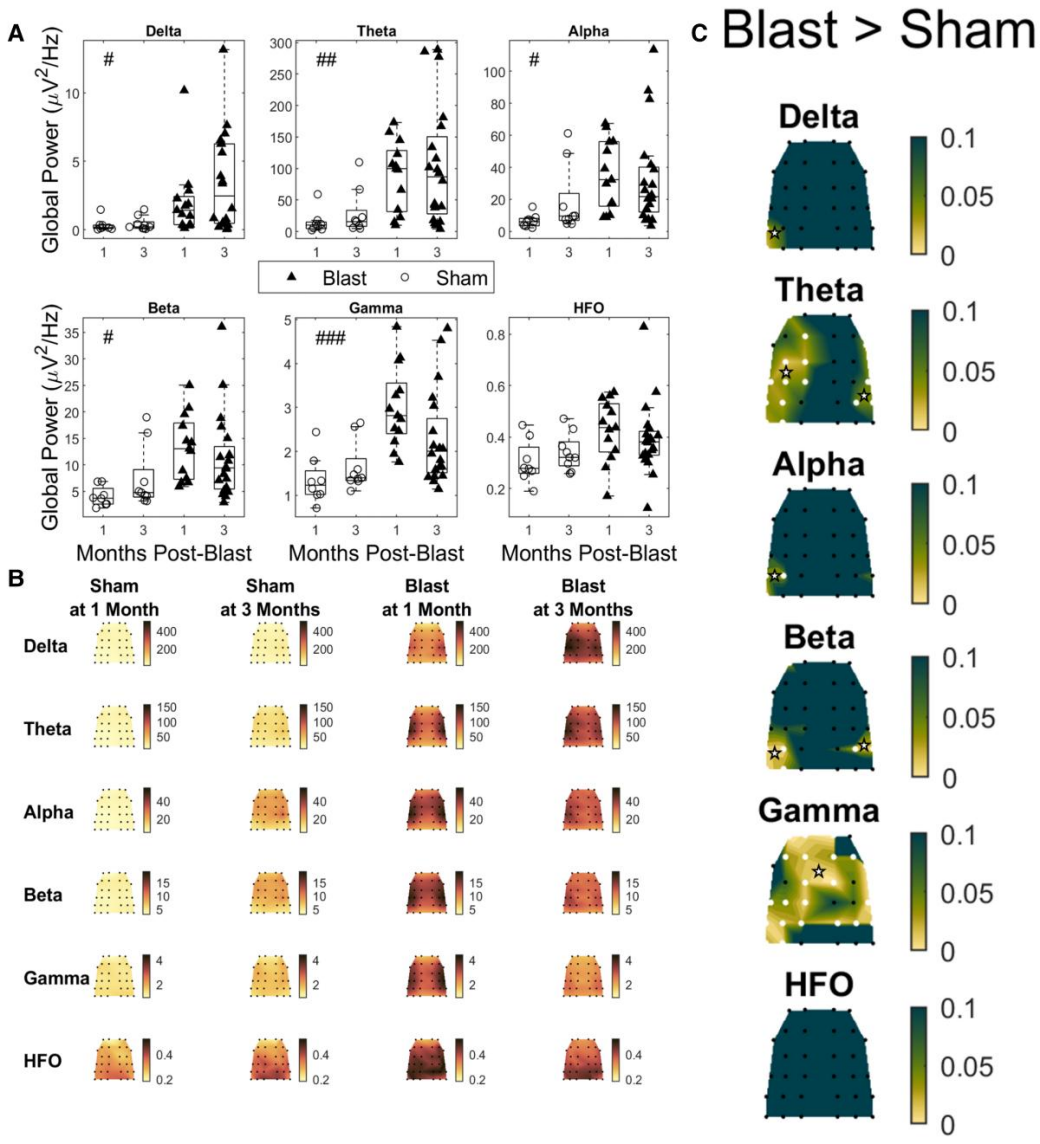
**Absolute power in blast and sham groups at 1- and 3-months post-injury.** (**A**) Global absolute power for all groups across all frequency bands. Delta: 1–4 Hz, theta: 4–8 Hz, alpha: 8–12 Hz, beta: 12–25 Hz, gamma: 25–80 Hz and HFO: 80–200 Hz. Permutation-based 2-way ANOVA (Group × Time post-blast with interaction, 10 000 iterations) with Bonferroni correction for multiple comparisons (*n* = 6 bands). Significant main effects of group were found for all frequency bands except for the HFO band. Significance values for all tests #: *P*-adj < 0.05 main effect of group, ##: *P*-adj < 0.01 main effect of group, ###: *P*-adj < 0.001 main effect of group, (**B**) Topoplots depicting mean group absolute power for all groups and frequency bands, across the multi-electrode array. (**C**) Topoplots depicting *P*-values for the main effect of group (permutation-based 2-way ANOVA, 10 000 iterations), Bonferroni corrected for multiple comparisons (*n* = 32 channels) for all bands. Channels for an adjusted *P*-value below 0.05 are marked with white stars (blast 1-month: *n* = 13, blast 3-month: *n* = 20, sham 1-month *n* = 8, sham 3-month *n* = 9).

Analysis of ‘absolute’ global EEG power revealed a main effect of injury in EEG power (Fig. 3A) (blast versus sham; all cases: two-way ANOVA; *F*(1,46)) with significantly elevated power in the blast groups for most bands examined (delta: *P* = 0.0138, theta: *P* = 0.0054, alpha: *P* = 0.0222, beta: *P* = 0.0172 and gamma: *P* = 0.0006).

To further localize the effect, we tested the main effect of injury (all cases: two-way ANOVA; *F*(1,46)) at the electrode level for all the frequency bands of interest (Fig. 3B and C). An increase in power in the delta band was observed for the blast group in the temporal region of the side contralateral (left) to the blast, as well as in the theta band in temporal, medial and frontal electrodes of the left hemisphere, and in the temporal electrodes of the right hemisphere. Power increases in the left temporal region were also observed in the alpha band for the blast group. In the beta band, the blast group had increased power along the left and right temporal regions. Finally, in the gamma band, the blast group displayed increased power in medial, temporal and frontal regions in both hemispheres. No spatially localized effect of injury was detected for the HFO band.

**Figure 4:**
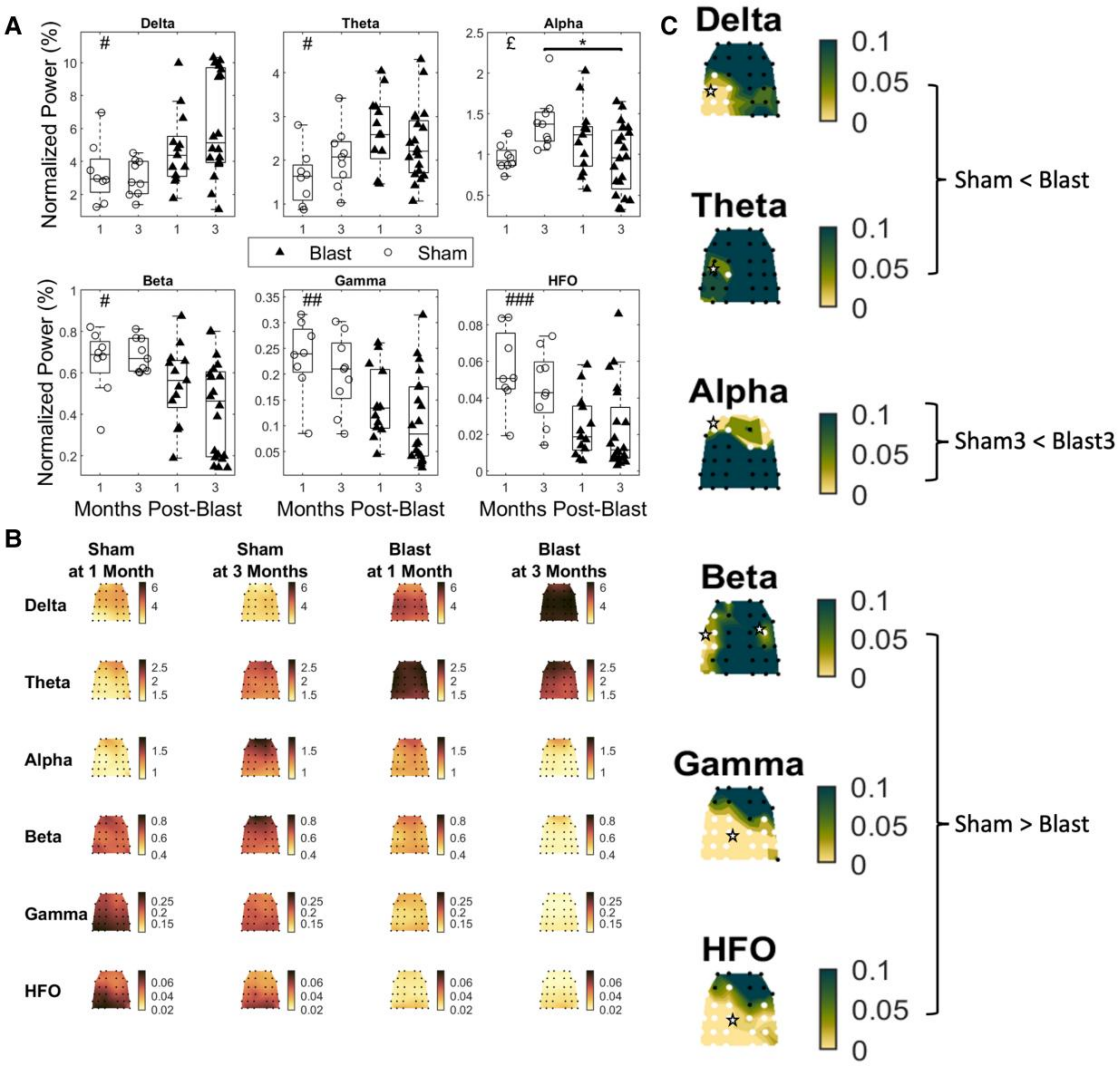
**Normalized power in blast and sham groups at 1 and 3-months post-injury.** (**A**) Global normalized power for all groups across all frequency bands. Delta: 1–4 Hz, theta: 4–8 Hz, alpha: 8–12 Hz, beta: 12–25 Hz, gamma: 25–80 Hz and HFO: 80–200 Hz. Permutation-based 2-way ANOVA (Group × Time post-blast with interaction, 10 000 iterations) with Bonferroni correction for multiple comparisons (*n* = 6 bands). Significant main effects of group were found for all frequency bands except for the alpha band. Significant interaction in the alpha band was followed by pairwise permutation-based *t*-tests (10 000 permutations) between the groups of interest with Bonferroni correction for multiple comparisons (*n* = 3 pairs). Significance values for all tests #: *P*-adj < 0.05 main effect of group, ##: *P*-adj < 0.01 main effect of group, ###: *P*-adj < 0.001 main effect of group, £: *P*-adj < 0.05 interaction effect ⋆: *P*-adj < 0.05 two sample *t*-test. (**B**) Topoplots depicting mean group normalized power for all groups and frequency bands, across the multi-electrode array. (**C**) Topoplots depicting *P*-values for the main effect of group (permutation-based 2-way ANOVA, 10 000 iterations), Bonferroni corrected for multiple comparisons (*n* = 32 channels) for the delta, theta, beta, gamma and HFO bands. The topoplot for the alpha band depicts *P*-values for the two-sample *t*-test between sham at 3-months and blast at 3-months. Channels for which the adjusted *P*-value is below a threshold of 0.05 are marked with white stars (blast 1-month: *n* = 13, blast 3-month: *n* = 20, sham 1-month: *n* = 8 and sham 3-month: *n* = 9).

Analysis of global ‘normalized’ EEG power (Fig. 4A) revealed a significant main effect of injury (blast versus sham; all cases: two-way ANOVA; *F*(1,46)) in most bands examined, driven by significantly elevated power after blast in the low frequency (delta: *P* = 0.0132, theta: *P* = 0.0498) and decreased power for the high frequency bands (beta: *P* = 0.015, gamma: *P* = 0.003 and HFO: *P* < 0.001). In the alpha band, a significant interaction was detected (*F*(1,46), *P* = 0.0246), and pairwise comparisons (unequal variances *t*-test; degrees of freedom: *ν* = 18.9076, calculated using Satterthwaite's approximation) revealed a significant decrease in power for the blast group at 3-months compared to the sham group at 3-months (*P* = 0.0105). No interaction was observed in any of the other bands considered.

To further localize the effect, we performed testing for the main effect of injury group (all cases: two-way ANOVA; *F*(1,46)) at the electrode level for all the frequency bands of interest and testing for the difference between the sham and blast groups at 3-months for the alpha band (unequal variances *t*-test; degrees of freedom: *ν* = 18.9076, calculated using Satterthwaite's approximation) (Fig. 4B and C). For the delta band, normalized power was increased (*P <* 0.05) for the blast group in a region covering the temporal and medial region of the left hemisphere. Normalized power was also elevated for the theta band for the blast group in the left medial region. For the alpha band, the blast group at 3-months had reduced normalized power in frontal regions compared to the sham group at 3-months. In the beta band, the blast group had reduced normalized power in locations along the left temporal and parietal regions. Finally, for the gamma and HFO bands, the pattern was similar, with the blast group having widespread decreased normalized power in posterior regions, spanning medial and temporal regions for both bands and an additional left frontal region for the HFO band.

### Mild blast injury increases FC in the gamma band at chronic time points

**Figure 5:**
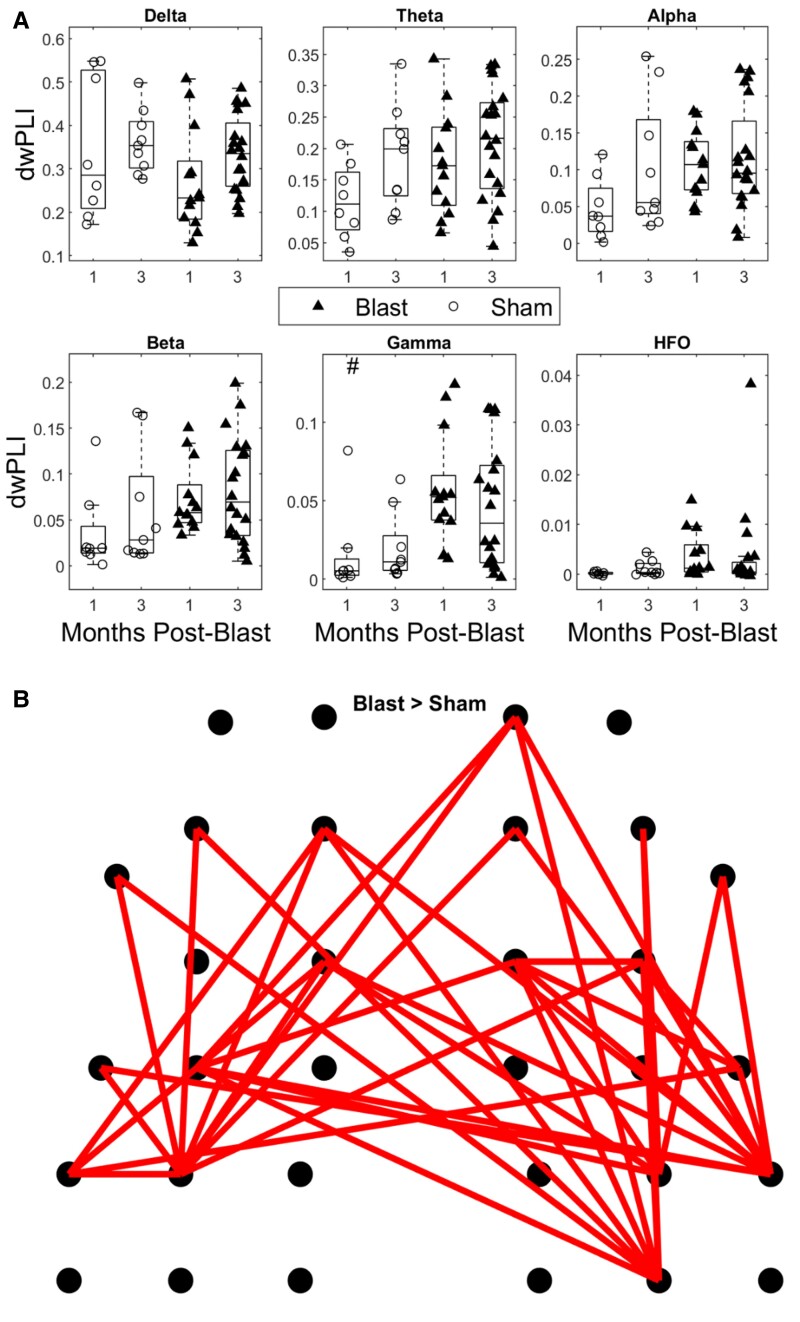
**Phase-based FC (dwPLI) in blast and sham groups at 1- and 3-months post-injury.** (**A**) Global connectivity for all groups. Permutation-based 2-way ANOVA (Group × Time post-blast with interaction, 10 000 iterations) with Bonferroni correction for multiple comparisons (*n* = 6 bands). A significant main effect of group was found only for the gamma band. Significance values for all tests ⋆: *P*-adj < 0.05. (**B**) Topoplot depicting a significantly hyperconnected network for the blast group. Independent-samples *t*-test (blast versus sham) based on 50 000 permutations with an FDR-adjusted threshold of *P* = 0.05, using the Network-based statistic toolbox (blast 1-month: *n* = 13, blast 3-month: *n* = 20, sham 1-month: *n* = 8 and sham 3-month: *n* = 9).

Analysis of global connectivity for all bands of interest (Fig. 5A, all cases: two-way ANOVA; *F*(1,46)), revealed significantly elevated values for the blast group specific to the gamma band (injury group effect *P* = 0.0108). After pooling the 1- and 3-months datasets for both injury groups and conducting further analysis at the edge level for the gamma networks, a robust network (one-sided independent samples *t*-test; *t* (49), *P* < 0.05) of increased connectivity in the blast group was obtained (Fig. 5B). This network included both interhemispheric and intrahemispheric connections spanning frontal, central, temporal and medial regions.

### Mild blast exposure decreases layer and region-specific PV and SST interneuronal density at 1 month and 3 months post-blast

**Figure 6:**
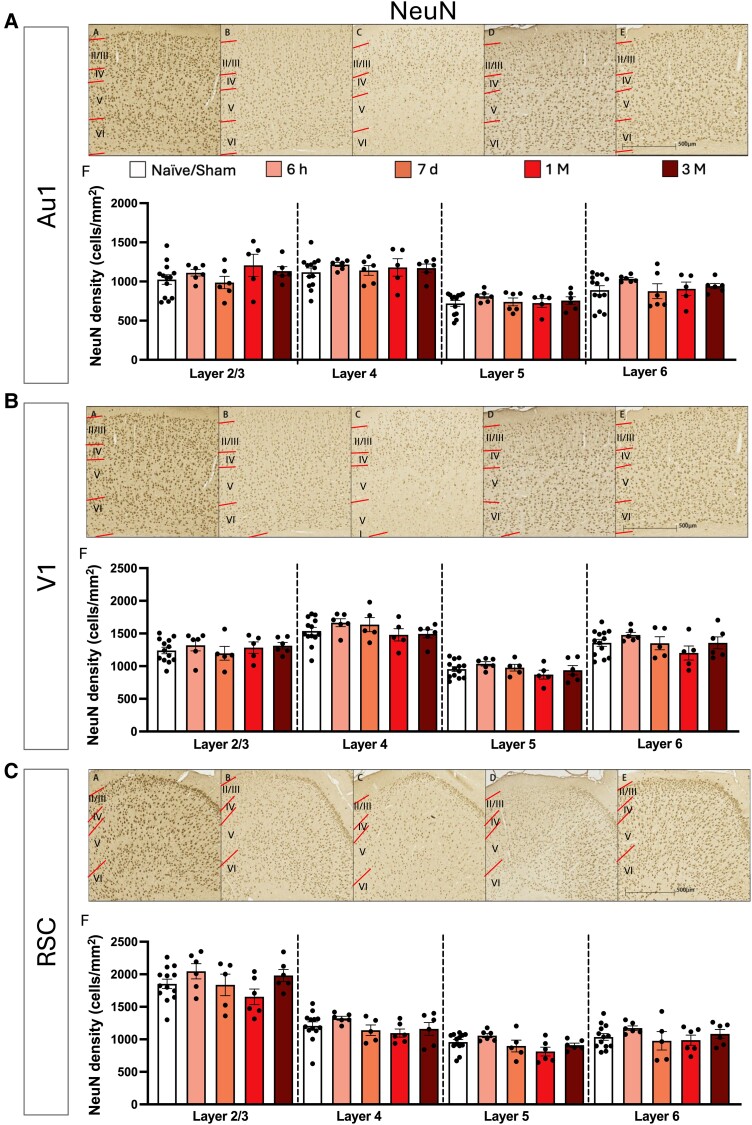
**NeuN density changes in the primary auditory cortex, the primary visual cortex and the RSC post-bTBI.** Micrograph of all cortical layers (denoted by upper case Roman numerals, layer boundaries indicated with red lines). Micrograph of all cortical layers in the primary auditory cortex (Au1) (**A**) for (AA) Naïve/Sham, (AB) 6-h post-injury, (AC) 7-days post-injury, (AD) 1-month post-injury and (AE) 3-months post-injury. (AF) Density of NeuN positive cells following single bTBI in the Au1 for each layer. Micrograph of all cortical layers in the primary visual cortex (V1) (**B**) for (AB) Naïve/Sham, (BB) 6-h post-injury, (BC) 7-days post-injury, (BD) 1-month post-injury and (BE) 3-months post-injury. (BF) Density of NeuN positive cells following single bTBI in the primary V1 for each layer. Micrograph of all cortical layers in the RSC (**C**) for (CA) Naïve/Sham, (CB) 6-h post-injury, (CC) 7-days post-injury, (CD) 1-month post-injury and (CE) 3-months post-injury. (CF) Density of NeuN positive cells following single bTBI in the RSC for each layer. Quantification of NeuN cell density using one-way ANOVA, corrected using two-stage linear step-up procedure of BKY FDR approach with *Q* = 5%. All data presented mean ± SEM. *P < 0.05, **P < 0.01, ***P < 0.001 and ****P < 0.0001 (Au1: Naïve/Sham: *n* = 13, 6 h: *n* = 6, 7-day: *n* = 6, 1-month: *n* = 5, 3-months: *n* = 6. V1: Naïve/Sham: *n* = 13, 6 h: *n* = 6, 7-day: *n* = 5, 1-month: *n* = 5, 3-months: *n* = 6. RSC: Naïve/Sham: *n* = 13, 6 h: *n* = 6, 7-day: *n* = 5, 1-month: *n* = 6, 3-months: *n* = 6).

We performed NeuN staining as a marker of neurons to determine whether electrophysiological changes were associated with alterations in gross scale cortical neuronal density. Our results revealed no effects on overall neuronal number in the ROIs (Fig. 6).

**Figure 7:**
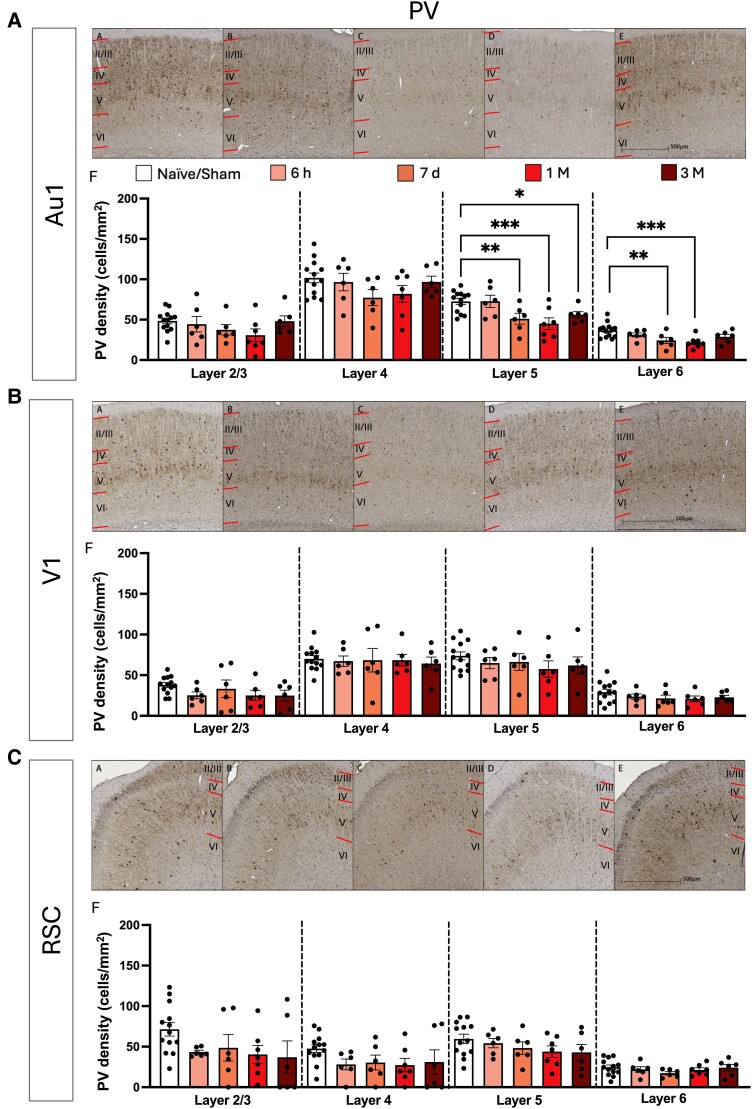
**PV density changes in the primary auditory cortex, the primary visual cortex and the RSC following blast TBI.** Micrograph of all cortical layers (denoted by upper case Roman numerals, layer boundaries indicated with red lines). Micrograph of positively stained PV interneurons in all cortical layers in the primary auditory cortex (Au1) (**A**) for (AA) Naïve/Sham, (AB) 6-h post-injury, (AC) 7-days post-injury, (AD) 1-month post-injury, (AE) 3-months post-injury. (AF) Density of PV positive cells following single bTBI in the Au1 for each layer. Micrograph of all cortical layers in the primary visual cortex (V1) (**B**) for (BA) Naïve/Sham, (BB) 6-h post-injury, (BC) 7-days post-injury, (BD) 1-month post-injury and (BE) 3-months post-injury. (BF) Density of positive PV interneurons following single bTBI in the primary V1 for each layer. Micrograph of all cortical layers in the RSC (**C**) for (CA) Naïve/Sham, (CB) 6-h post-injury, (CC) 7-days post-injury, (CD) 1-month post-injury, (CE) 3-months post-injury. (CF) Density of PV interneurons following single bTBI in the RSC for each layer. Quantification of PV interneuron cell density was conducted using one-way ANOVA, corrected using Two-stage linear step-up procedure of BKY FDR approach with *Q* = 5%. All data presented mean ± SEM. *P < 0.05, **P < 0.01, ***P < 0.001 and ****P < 0.0001 (Au1: Naïve/Sham: *n* = 13, 6 h: *n* = 6, 7-day: *n* = 6, 1-month: *n* = 7, 3-months: *n* = 6. V1: Naïve/Sham: *n* = 13, 6 h: *n* = 6, 7-day: *n* = 6, 1-month: *n* = 7, 3-months: *n* = 6. RSC: Naïve/Sham: *n* = 13, 6 h: *n* = 6, 7-day: *n* = 6, 1-month: *n* = 6, 3-months: *n* = 6).

We then investigated whether specific neuronal types might be affected by blast exposure. As mentioned, PV and SST interneurons have been shown to be preferentially damaged by TBI and were therefore selected for histological analysis. GABAergic PV INs were analysed in three cortical regions and only the fifth and sixth layer of the Au1 was found to be significantly affected (layer 5: *F*(4, 33) = 5.291, *P* = 0.0021, Benjamini, Krieger and Yekutieli (BKY) false discovery rate (FDR) adjusted-*P* value: *q* = 0.0084, layer 6: *F*(4, 33) = 4.670, *P* = 0.0043, *q* = 0.0172: Fig. 7A). Further analysis revealed a significant decrease in PV INs density at 7-days and 1-month post-injury for both the fifth (7-days: *P* = 0.0084, 1-month: *P* = 0.0006, a reduction of 29.53% for 7-days and 45.73% for 1-month post-injury relative to control) and sixth cortical layers (7-days: *P* = 0.0041, 1-month: *P* = 0.0005, a reduction of 33.88% and 47.63%, respectively). There were no significant changes in PV IN density in any cortical layer for both the V1 (Fig. 7B) and the RSC (Fig. 7C), *P* and *q* > 0.05.

**Figure 8:**
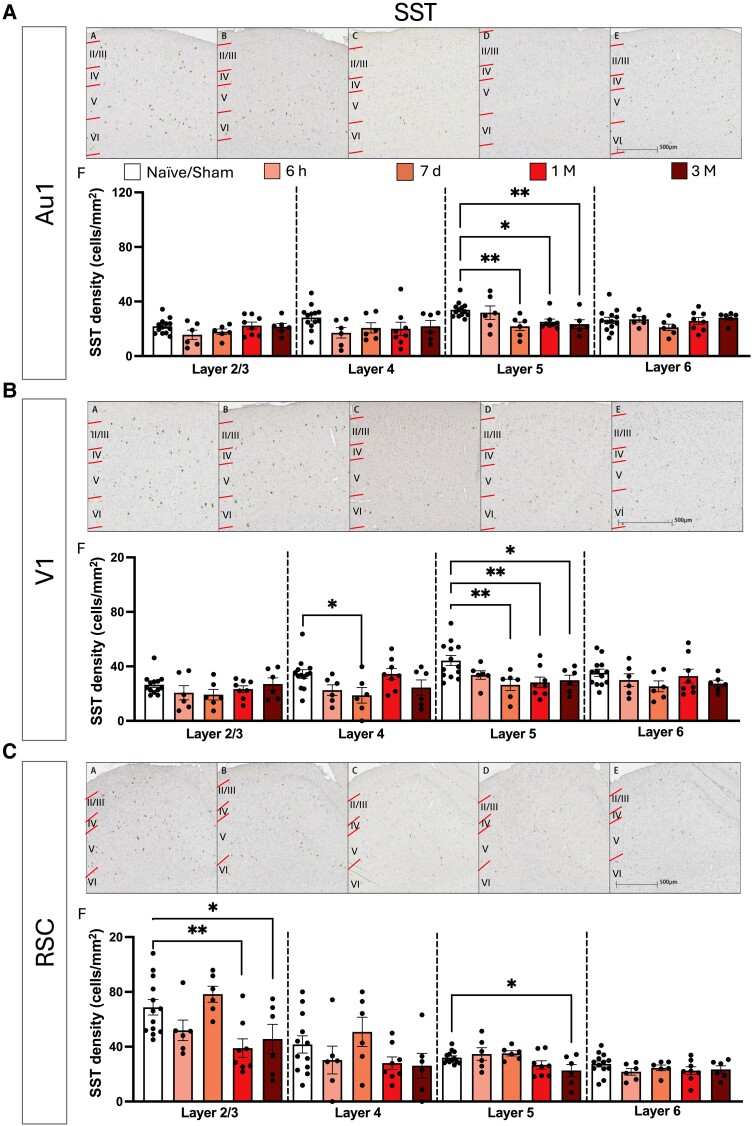
**SST density changes in the primary auditory cortex, the primary visual cortex and the RSC post-bTBI**. Micrograph of all cortical layers (denoted by upper case Roman numerals, layer boundaries indicated with red lines). Micrograph of positively stained SST interneurons in all cortical layers in the primary auditory cortex (Au1) (**A**) for (**A**)A Naïve/Sham, (AB) 6-h post-injury, (AC) 7-days post-injury, (AD) 1-month post-injury and (AE) 3-months post-injury. (AF) Density of SST positive cells following single bTBI in the Au1 for each layer. Micrograph of all cortical layers in the primary visual cortex (V1) (**B**) for (BA) Naïve/Sham, (BB) 6-h post-injury, (BC) 7-days post-injury, (BD) 1-month post-injury and (BE) 3-months post-injury. (BF) Density of positive SST interneurons following single bTBI in the primary V1 for each layer. Micrograph of all cortical layers in the RSC (**C**) for (CA) Naïve/Sham, (CB) 6-h post-injury, (CC) 7-days post-injury, (CD) 1-month post-injury and (CE) 3-months post-injury. (CF) Density of SST interneurons following single bTBI in the RSC for each layer. Quantification of SST interneuron cell density was conducted using one-way ANOVA, corrected using two-stage linear step-up procedure of BKY FDR approach with *Q* = 5%. All data presented mean ± SEM. *P < 0.05, **P < 0.01, ***P < 0.001 and ****P < 0.0001 (Au1: Naïve/Sham: *n* = 13, 6 h: *n* = 6, 7-day: *n* = 6, 1-month: *n* = 8, 3-months: *n* = 6. V1: Naïve/Sham: *n* = 13, 6 h: *n* = 6, 7-day: *n* = 6, 1-month: *n* = 8, 3-months: *n* = 6. RSC: Naïve/Sham: *n* = 13, 6 h: *n* = 6, 7-day: *n* = 6, 1-month: *n* = 8, 3-months: *n* = 6).

The density of SST positive cells in the fifth layer of the neocortex was found to be significantly reduced at specific time points post-injury. One-way ANOVAs, corrected using previously mentioned multiple-comparison correction method, revealed significant differences in the fifth layer in the Au1 (*F*(4, 34) = 4.140, *P* = 0.0077, *q =* 0.024), V1 (*F*(4, 34) = 4.241 *P* = 0.0068, *q =* 0.0214) and RSC (*F*(4, 34) = 3.069, *P* = 0.0292, *q* = 0.0460). Further analysis showed that densities decreased from 7-days post-injury relative to Naïve/Shams in the Au1 (Fig. 8A) (7-days: *P* = 0.0029, 1-month: *P* = 0.0147, 3-months: *P* = 0.0085, and amounted to a reduction of 35.52% for 7-days, 25.89% for 1-month, and 30.91% for 3-months). Similar changes were observed in the V1 with density decreases from 7-days post-injury (Fig. 8B) (7-days: *P* = 0.0025 1-month: *P* = 0.0029, 3-months: *P* = 0.0124, which constitutes a density reduction of 40.34%, 36.09% and 32.55% respectively). A *post hoc* test revealed a significantly lower cell density at 3-months post-injury in the fifth layer of the RSC compared to control (*P =* 0.0201, a 29.47% reduction relative to Naïve/Sham). A significant decrease in cell density was found in 2/3rd layer of the RSC (*F*(4, 34) = 5.010 *P* = 0.0028, *q* = 0.0088) with 1-month and 3-months post-injury driving the change (1-month: *P* = 0.0022, 3-months: *P* = 0.0252, a density reduction of 43.35% and 33.61%, respectively relative to Naïve/Sham) (Fig. 8C). No significant differences were found in other layers of the other cortical ROIs (*P* and *q* > 0.05).

### Mild blast exposure causes microglial changes at acute and chronic time points

To examine whether an inflammatory response accompanied the PV and SST loss after blast exposure, glia densities (IBA1- and GFAP-positive cells) were assessed in the same cortical areas. Significant increases in microglia density were observed in the Au1 (*F*(4, 27) = 4.392, *P* = 0.0073) and RSC (*F*(4, 27) = 5.504 *P* = 0.0023), but not the V1 or the CC (*P >* 0.05). Further analysis revealed an increase of 34% at 6-h (*P* = 0.0004) and 18% at 1-month post-injury (*P* = 0.0414) for the Au1 driving the change in IBA1 density, while for the RSC significant differences were found at 6-h (40% increase *P* = 0.0001), 7-days (25% increase, *P =* 0.0207) and 1-month (20% increase *P* = 0.0288) compared with Naïve/Sham animals. Despite the non-significant main effect for the other regions, there was a trend of increased IBA1 density at 6-h in the CC and RSC (Supplementary Fig. S2).

Results for percentage IBA1 area covered was found to be significant in the AU1, (*F*(4,26 = 2.983), *P* = 0.0417), V1 (*F*(4,26 = 3266), *P* = 0.0269), and CC (*F*(4,26 = 5.324, *P* = 0.0029), with only borderline differences in the RSC (*F*(4, 26) = 2.374, *P* = 0.0782). *Post hoc* analysis revealed that there was an increase in the IBA1 percentage area stained at chronic time points post-injury in all brain areas investigated, as shown in Supplementary Fig. S3 (Au1: area increase of 96.82%, *P* = 0.0326, V1: 106.43% increase, *P* = 0.0250, CC: 266.38% increase, *P* = 0.0006) and the RSC followed the same increasing trend at this chronic time point (Supplementary Fig. S3).

GFAP area covered as measurement of reactive astrogliosis showed no significant increases in the Au1, V1, RSC and CC (*P* > 0.05) (Supplementary Fig. S4).

### Mild blast injury does not affect CC integrity

NFL is a fundamental neuronal scaffolding protein and has been shown to indicate axonal damage and neurodegeneration in TBI and other neurodegenerative diseases.^68^ However, in this study no significant changes were observed in the CC (Supplementary Fig. S5).

## Discussion

This study explores the use of high-density EEG, as a clinically translatable diagnostic tool in an animal model of mild bTBI. This is the first study to combine cellular pathology, multi-electrode high-density EEG recordings at the whole brain level, and changes in SST interneuron cell density over multiple time points, from the acute to chronic phases post-injury.

Our findings indicate that a single mild blast injury can result in chronic global increases in power and increased gamma band FC. These electrophysiological signatures of mild bTBI were accompanied by significant non-focal decreases in key inhibitory neural population densities over time. Specifically, there was a decrease in SST interneuron density in layer 5 across all cortical ROIs, and a reduction in PV interneuron density in layer 5 of the primary auditory cortex. Additionally, we observed a mild inflammatory response following blast.

We only observed minimal effects of blast on electrophysiological metrics of brain activity at the acute time point. The time of recording (3–4 h post-injury) is consistent with the literature indicating that the EEG signal returns to baseline 1-h after injury (see^69^ for a review of the time course of EEG changes after TBI). This suggests that the chronic changes we observed could be a result of mechanisms acting over longer timescales, likely related to secondary injury mechanisms. This also indicates the potential availability of a therapeutic window at this stage.

However, we observed significantly elevated FC in the HFO band, where activity in an overlapping frequency band has been associated with epileptogenesis^70,71^ and could occur as part of the pathological cascade driven by hyperexcitability.^71,72^ In our model, this could be driven by excessive release of excitatory neurotransmitters and accumulation of calcium, both well-documented stages in the post-injury cascade.^73^ This mechanism may also drive the increase in inflammatory markers^74,75^ observed 6-h post-injury^74^ (Supplementary Fig. S2), potentially establishing a positive feedback loop of hyperexcitability and neuroinflammation.^76-78^ Nevertheless, given our observed changes in connectivity rather than activity and the lack of behavioural data, the hypothesis of epileptogenic dynamics in our injury model requires further exploration in future studies. These studies should also explore localized effects of injury in the acute cohort that could be obscured by our analysis at the global level.

Hyperexcitability and an imbalance in excitation and inhibition are persistent sequelae of TBI. Extra-cranial electrophysiology has been proposed as a tool to monitor these changes in neuronal functionality.^79^ Most human studies quantify EEG in terms of ‘relative’ (normalized to total subject power) power changes.^37^ This is to account for the large degree of individual variability found in total power in human EEG studies.^41^ While this approach is understandable, it will miss important patterns of effects such as a uniform increase in power across all frequencies. Due to the reduction of individual variability available in a preclinical laboratory environment, our model was able to add increased explanatory power to clinically relevant findings by including absolute measures of change. Our analysis of relative power (Fig. 4) yielded similar results to human studies,^37^ showing a relative increase in low frequencies and a decrease in the higher frequencies. However, we also demonstrated that this was accompanied by a broadband increase in absolute power (Fig. 3). This suggests the presence of hyperexcitability in our mild bTBI model during the chronic post-injury phase, as similar patterns of increased power have been observed in a knockout model of Fragile-X syndrome,^80^ a condition linked to circuit hyperexcitability.^81^ Crucially, the two analysis approaches converge in terms of increases in low frequency power, which is a hallmark signature of TBI and can be interpreted more confidently.^29,37^ Additionally, we have identified an absolute measure of power change as a possible EEG signature of neural hyperexcitability.

Several human studies have established an increase in power in lower frequencies as a hallmark electrophysiological signature of TBI.^29,82-85^ This has been linked to cognitive impairment^37^ and structural disconnection due to axonal injury and white matter damage.^29^ In our results, we show this same typical electrophysiological signature (Figs. 3 and 4); however, this was not accompanied by indicators of white matter damage (NFL changes) in the CC (Supplementary Fig. S5). These findings are contrary to the minimal NFL histology data in the TBI modelling field that are typically characterized by more focal and severe injuries.^68,86^ Notably, there is a dearth of studies investigating NFL using histology specifically in ‘blast’ induced TBI, making direct comparisons challenging. However, a study on mild blast TBI in rats observed elevated levels of SMI-31 (neurofilament heavy and therefore an indicator of white matter damage) at 3 DPI,^87^ a time point not assessed in our research. Consequently, it is plausible that the observed pathology had occurred after our 6-h time point and was then resolved by our 7-day time point.

In line with recent efforts to boost the translational value in preclinical models of neurological disease and align them with modern human electrophysiology studies,^88,89^ we used a high-density EEG array to record brain activity. This allowed us to explore electrophysiological correlates of blast TBI both on a global and local scale. With this, we were able to identify regionalized effects of absolute power for the delta, theta, alpha and beta bands in posterior temporal regions, while for the gamma band the effect was more widespread across the array. For relative power, effects for the delta, theta, beta and gamma bands were localized in a similar pattern. Alpha band effects were concentrated in frontal areas, with HFO band effects widespread across central and posterior regions. This pattern of results lends an added degree of spatial sensitivity to our analysis, allowing detection of region-specific signatures of injury and their putative asymmetries across cortex. Future studies could explore the causes of these changes by studying the injury biomechanics through computational modelling,^87^ and take this spatial heterogeneity into account when designing therapeutic interventions.

FC studies in TBI show inconsistent findings^35,37,90^ and this extends to the small number of studies that have investigated FC changes in blast cohorts. Sponheim *et al*.^91^ and Wang *et al*.^42^ both found reduced FC due to blast but were inconsistent regarding the frequency bands where this reduction was found. In contrast to other studies, and in line with our findings, Huang *et al*.^92^ reported an ‘increase’ in FC in a human study and found this in the gamma and beta frequency bands. One suggested reason for this seemingly conflicting finding was that subjects in the Huang study were more likely to have suffered a ‘milder’ form of injury and this may have triggered a different pathological path to that found in the more severe injury types typically studied. Indeed, our similar finding in this animal model of ‘mild’ bTBI lends support to this hypothesis.

‘Functional hyperconnectivity’ has been observed to continue from the acute (24 h; Manning^93^) to the chronic phase (2.5 years on average but up to 6.6 years; Sharp^94^) post-TBI injury, suggesting a protracted elevated shift in baseline connectivity. This is consistent with our results showing hyperconnectivity up to 3-months post-injury in rats, roughly corresponding to 1–9 years post-injury in ‘human time’.^95^ An increase in FC as a response to TBI has been correlated to cognitive performance with evidence in favour of it being an adaptive phenomenon,^94,96-98^ with the exact relationship to cognition likely depending on injury type, time point of assessment, methods, region/network and cognitive demand (e.g. see the mixed correlations with behavioural measures in Huang^92^). An intriguing interpretation suggests that increased transmission across regions might be a compensatory effect associated with an increase in the efficiency of information exchange across the cortex.^99^ This increase in connectivity could be an attempt to maintain adequate signal to noise ratios, as seen in aging,^100^ in response to the injury.^100,101^ However, it is possible that this hyperexcitability in response to injury could also be a risk factor for the development of future neurodegenerative disease. Indeed, a link has already been established between early adult hyperconnectivity in the gamma range (40–160 Hz) and an increased genetic risk of developing dementia.^102^

In the healthy brain, local cortical gamma oscillations are generated by mutual inhibitory loops within PV cell populations as well as inhibitory-excitatory loops between PV and pyramidal neurons.^103,104^ Given the critical involvement of PV interneurons in the generation of gamma-band activity,^105^ the increase in specifically gamma band FC could suggest a circuit-level mechanism as the substrate of our observed hyperconnectivity. In addition to targeting distant-region pyramidal neurons, cortical pyramidal cells in layer 5 provide excitatory inputs to inhibitory VIP neurons.^106^ These VIP neurons inhibit a target region’s SST neurons, and by doing so effectively disinhibit both pyramidal and PV neurons in that target region’s gamma generating circuit.^107-111^ In our model of bTBI, it is proposed that SST neuronal loss interferes with this mechanism and leads to a disinhibited flow of gamma oscillations to the target region, manifesting as increased FC in the gamma range. In addition, enhanced plasticity due to injury-related hyperexcitation could exacerbate this phenomenon by creating stronger synapses and thus more reliable transmission of the oscillations by pyramidal neurons.^48,112^ Importantly, studies investigating other pathologies associated with an imbalance in excitation and inhibition such as Fragile X syndrome,^41^ autism spectrum disorder^113^ and schizophrenia^114,115^ have reported the same finding of increased gamma connectivity. Thus, our results provide a potential mechanism mediating post-traumatic connectivity increases, which could in turn underlie cognitive symptoms and provide a therapeutic target for intervention.

When considering the impact of blast on electrophysiological measures such as FC, it is important to consider accompanying neuronal and structural changes. Changes in NeuN, a measure of overall neuron density, can be observed in more severe models of TBI such as experimental CCI models.^116-118^ However, in the current study, and consistent with previous research of single-event mild TBI,^119^ NeuN staining indicated no significant change in overall neuron density in the ROI. Before drawing a conclusion of no neuronal loss, it must be considered that NeuN measurements have possible limitations when identifying interneuronal sub-type density changes. For example, a 40% loss of an interneuron population such as PV or SST, would only result in a ∼2% change in overall neuronal density when using NeuN. As a result, a lack of change in NeuN staining possibly only reflects the gross impact of blast on all neurons in a region. To counter this potential limitation of NeuN staining, we separately evaluated the neuronal density of two of the most abundant interneuron subtypes in the brain, PV and SST, which have been repeatedly implicated in TBI pathology.^48,57,120,121^ It has been further demonstrated that PV interneuron populations are more vulnerable to non-blast induced TBI than SST interneurons.^49^ In contrast to more severe models of TBI, we found SST interneurons to be more affected by a mild blast injury than PV interneurons. However, as with previous research, we found the fifth layer to be most vulnerable to injury.^48^

When evaluating immunopositive staining results, conclusions regarding decreases in SST across all ROI contain an element of ambiguity, as they could be due to interneuron loss and/or downregulation of neuropeptide SST (which is not staining positive). For example, it has been shown in a more severe injury model that SST interneuron density decreases after non-blast TBI.^48^ As with SST density, a decrease in immunopositively stained PV interneurons can indicate either PV interneuron loss or a reduction in the density of PV by these neurons.^56^ In this single blast model of mild TBI, only PV interneurons in the primary auditory cortex were affected, but this might be a result of hearing impairment or loss rather than the blast itself.^120^ This could be the case in our model since no ear protection was used in our experimental setup and could have led to temporary hearing loss due to eardrum rupture. Given that there was no interneuronal recovery over any time points post-injury, this could also suggest our results are showing interneuron cell death rather than down-regulation of their expression. Nevertheless, whether our results are an indication of interneuron loss or downregulation of the expression, or both, it could disrupt the E/I imbalance and cause neural hyperexcitability.^43,122^

In addition, our data show an immediate microglial density increase post-injury as a sign of inflammatory activation, which lasts up until 1-month post-injury in the Au1 and RSC. Our results also demonstrate an increase in percentage area stained in all cortical regions other than the RSC at 3-months post-injury, indicating hypertrophy and potentially a change in phenotype. This result aligns with other acute time point studies of blast,^123^ and mild blast TBI studies that see both inflammatory responses at later time points.^3,120,124,125^ Finally, our results show that there was no significant increase in astrocytic activation at any time point after injury, thus suggesting no obvious astrocyte influence on the electrophysiological changes seen in our study.^126-128^ It is noteworthy that microglial activation occurs without astrocytic activation, as both cell types are crucial responders to injury and release cytokines to communicate with each other.^129^ Our findings contrast with some previous studies that report astrocytic activation, particularly at acute time points post-injury.^126-128^ However, astrocytic activation is not always defined by astrocytes expressing higher levels of GFAP. Activated astrocytes can, in the absence of significant GFAP expression, alter their functionality and morphology post-TBI. Additionally, it should be noted that other markers of astrocytic activation such as glutamine synthase and S100β might be more sensitive to these changes.^130,131^ However, a study that investigated bTBI with a similar impulse energy and subject orientation to the blast wave obtained similar findings to ours,^132,133^ possibly highlighting pathology heterogeneity due to different blast parameters in terms of determining the nature of injury outcomes. Furthermore, due to the diffuse nature of the injury in our model astrocyte activation was not observed in the cortex, but may still be present in other regions not investigated.

In this study, we have developed a preclinical animal model of mild blast-induced TBI and demonstrated that the employed techniques and metrics of injury assessment reflect the subtle changes associated with mild injury. As electrophysiology is already used in the clinical setting, our employed outcome measures are highly translatable. These findings are particularly relevant when considering multiple TBI lifetime events are a key risk factor for future neurodegeneration,^4^ which makes the identification of injury in the absence of preclinical symptoms vital. By obtaining EEG correlates of injury, clinicians will have an additional tool to identify a potentially vulnerable condition that would require someone to refrain from engaging in activities with a high risk of further brain injury, thus mitigating the risk of future disease, Indeed, we anticipate future preventative treatment protocols in subjects at high-risk of brain injury (such as contact-sports, active military and police personnel) where regular EEG scans are obtained. First to obtain baseline metrics, and then to observe any future changes that might suggest a potential TBI event. We have combined histological findings only accessible in an animal model with clinically relevant EEG to obtain further clarity on the relationship between the structural and electrophysiological changes observed in TBI. This shows the value of high-density EEG to assess injury, and therefore present this as a potential tool to be used for TBI diagnosis and management of treatment.

Future research would do well to investigate the long-term structural causes of bTBI-induced hyperconnectivity and hyperexcitability and connect them to behavioural markers of injury to determine their contributions to neurogenerative disease. Although animal welfare restrictions often prohibit inflicting injuries on awake subjects, robust awake EEG recordings are feasible^88^ and could therefore be used to investigate chronic time points after injury. In addition, despite evidence to suggest that observed gamma-band connectivity differences are robust to anaesthetic state,^134^ future work should be conducted to validate these findings. Furthermore, forthcoming research should also address the limitations of the current study in terms of the between-subjects experimental design. Adoption of a longitudinal within-subjects design could both bolster statistical power and provide a firm ground for understanding the trajectory of injury progression.

## Supplementary Material

fcae385_Supplementary_Data

## Data Availability

The data underlying this article cannot be shared publicly because of ongoing work. The data will be shared on reasonable request to the corresponding authors.
