# Supplementary material for: EEG hyperexcitability and hyperconnectivity linked to GABAergic inhibitory interneuron loss following traumatic brain injury
Source: Brain Commun. 2024 Nov 27;6(6):fcae385. doi: 10.1093/braincomms/fcae385 (PMC11600960; doi:10.1093/braincomms/fcae385)
Supplement: fcae385_Supplementary_Data [file fcae385_supplementary_data.pdf]

Supplementary Information

TRBL-CBIS shock tube

All blast experiments used the Royal British Legion Centre for Blast Injuries (TRBL-CBIS) shock tube at Imperial College London. The shock tube comprises three separate 1.22 m long tubes with an internal diameter of 59 mm that are bolted together with flanges. The first 1.22 m long tube acts as the driver section and connects to the following two tubes known as the driven section (Fig. 1A). The driver section was reduced to 7% capacity with use of a partitioning blanking plate and was separated from the driven section by replaceable 250 µm Mylar® diaphragms. This reduction was necessary to produce a Friedlander waveform (Supplementary Fig. 1A) [1-3]. A shock wave was generated by filling the 7% driver section with compressed air until a Mylar® diaphragm burst, releasing the air through the driven section. The shock wave propagated down the driven sections and towards the right side of the rat head. Reproducibility of the shock wave was monitored using piezoelectric pressure sensors (Dytran Instruments 2300V1, California, USA) that were flush with the inside wall of the shock tube to give in-tube incident shock wave pressure readings from sensor 2 (Supplementary Fig. 1B). Control experiments were conducted using a phantom rat head model (as seen in Supplementary Fig. 1C), and a third sensor (sensor 3) was substituted for the rat's head to characterise blast at this location.

Blast Procedure

Due to logistical constraints related to the experimental procedures and COVID-19 restrictions, we were unable to randomise the rats for our experiments. The study involved a combination of blast traumatic brain injury (TBI) and EEG measurements, which required careful coordination between the experimental days and the availability of the animals and the experimenters. Given these time constraints and logistical challenges, randomising the animals at different time points, such as 1-month and 3-months, would have been highly inefficient and impractical for the study. To avoid confounding effects of skill level differences between experimentalists, each part of the procedure was performed by the same experimentalist across all experiments.

Animals were weighed and then anaesthetised in an induction chamber with 5% isoflurane at 2.5 L/min. Once anesthetised, an animal was moved to a physiological monitoring platform (Harvard Apparatus) where anaesthesia was maintained at 1.5-2.5% using a 2 L/min flow rate delivered via a polyethylene nose cone. Analgesia with buprenorphine (0.05 mg/kg s.c.; Vetergesic, UK) was administered 30 mins prior to blast, and lubrication was placed over the eyes. A closed loop temperature control system with a rectal probe was used to maintain body temperature at 37 °C. In addition, breathing rate and blood oxygenation were also monitored using the platform. Once an animal was stable under anaesthesia (approx. 20 mins), it was moved into a protective shield on a platform fastened to the shock tube exit. The animal was placed perpendicular to the shock tube exit, with the snout positioned 4.5 cm from the shock tube exit (with right side of the animal closest to blast) and protruding 5cm from the exit of the protective shield (schematic in Fig. 1A). This placement allowed the body to be off-centre to the tube exit to prevent blast lung injury. Blood oxygenation continued to be monitored whilst the animal was on the blast platform and breathing rate was manually counted prior to blast.

The animal was fixed from the torso down in the prone position inside the protective tube whilst the head was free to move in response to the shock wave. Anaesthesia was delivered via the soft polyethylene nose cone which would then be expelled during blast. After blast exposure the animal was immediately removed from its protective shield and placed on a heat mat in a lateral recumbent position. Oxygen-enriched air was provided, and blood oxygenation levels were monitored during recovery. 3 mL of sodium chloride 0.18% and glucose 4% saline solution for infusion (Baxter) was administered. Blast characteristics are presented in supplementary Table 1. Loss of righting reflex (LORR) was measured from the time of the blast until an animal had all four paws touching the ground in the recovery cage. The loss of righting reflex was used as a proxy for loss of consciousness (LOC) as used in humans.

Once an animal had fully recovered, it was returned to an individual cage and monitored until the end of the experimental day when it was returned to a cage of four. Animals were given buprenorphine (mixed in Hartley's strawberry, 1:2 jelly/water, 0.3 mg/kg p.o.) and weighed twice a day for 3 days. This was followed by daily welfare checks based on a based on a scoring system [4] and weighing once a day for two weeks, and then once-a-week welfare checks until termination. Typical signs of TBI should develop on recovery from anaesthesia and over the subsequent 72 hours, a period covered by the monitoring scheme described above. Animals were considered to have mild TBI due a Morton and Griffiths welfare score of below 2, indicating no signs of pain, distress or weight-loss post-injury, together with no pulmonary blast lung damage induced mortality. If animals had a welfare score that exceeded 2 they would not be considered a mild blast TBI and be excluded from the study.

Humane endpoints were set as follows: Any animal experiencing a high level of distress or a reduction in body weight of or more than 20% would be humanely killed by a Schedule 1 method. If an animal displayed 2 or more of the following clinical signs, it would be killed by a Schedule 1 method: piloerection, hunched posture, reduced mobility, pall or, ocular or nasal discharge.

For the acute electrophysiological cohort, blast-exposure was identical to the chronic cohorts except for the anaesthetic protocol. More specifically, animals were anaesthetized using urethane as described in the 'Electrophysiological Recordings – Surgery' section of the supplementary methods. This anaesthetic protocol results in a stable anaesthetic depth and thus after baseline recordings of brain activity, animals were placed at the shock tube exit for the injury procedure. After injury, animals were transferred to a different room for post-injury recordings while still under terminal general anaesthesia.

High speed videos

High-speed videos were used as quality control to ensure the animals were receiving the blast in the same manner similar to Mishra et al. [5]. (Software used: Physlets Tracker 5.1.5 (project source of: Open Source Physics www.compadre.prg/osp)).

Tissue processing

PFA-fixed brains of the animals were blocked with a 3D printed brain matrix – and split into 9 2 mm blocks of tissue. All tissue processing was performed by the same experimenter. The 4th block (-3.6 to -5.6 mm from Bregma) containing the all regions of interest was embedded

in paraffin and serially sectioned (7 mm) on a microtome. Slices were mounted onto individual slides, with 3 slices used per stain, per animal and averaged .

Immunostaining

All immunostaining and analysis procedures were conducted by same investigator.

GFAP and NeuN

Sections were deparaffinised and rehydrated using xylene and decreasing concentrations of ethanol. The tissue was washed in 1x PBS permeabilised and endogenous peroxidase quenched using 1% Hydrogen Peroxidase (H2O2) in 1x PBS + 1% Triton-100-X (PBS-Tx) (w/v) for 30 mins, followed by another 1x PBS wash and then antigen retrieval in 0.01M Citrate Buffer (pH6) for 20 mins in a steamer. Sections were incubated overnight at 4°C with the primary antibody GFAP (polyclonal, Rabbit IgG, #Z0334, DAKO, Denmark, 2.9 g/L, 1:2000) or NeuN (polyclonal, mouse IgG, #MAB377, Millipore, 1 mg/mL, 1:1000).

On the following day Super Sensitive polymer-HRP (BioGenex) IHC detection system was applied according to manufacturer's protocol. The slides were then stained with 3,3′-Diaminobenzidine (DAB,GFAP: 2 min, NeuN: 90 seconds) dehydrated, and cover slipped.

PV and IBA1

Tissue preparation (deparaffinisation, rehydration, endogenous peroxidase quenching, antigen retrieval) was the same as described before. This was followed by blocking using 10% foetal bovine serum (FBS), 10% Normal Goat Serum (NGS) and 1% Bovine Serum Albumin (BSA) for 2 hours. Slides were incubated overnight at room temperature with the primary antibody IBA1 (polyclonal, rabbit IgG, Fujifilm Wako Pure Chemical Corporation, #019-19741, 1:1000) and overnight at 4°C PV (polyclonal, rat IgG, #PV-235, Swant, 1:10,000) in 2%FBS/2%NGS/0.2%BSA PBS + 1% Triton-100-X (PBS-Tx) (w/v).

The following day, slides were incubated with the biotinylated secondary antibody (goat anti-rabbit biotinylated, #BA-1000, Vector Labs, 1:250, or Goat anti-rat biotinylated #BA-9400, Vector Labs, 1:250) in 2%FBS/2%NGS/0.2%BSA PBS + 1% Triton-100-X (PBS-Tx) (w/v) for 90 mins. The slides were then incubated with avidin-biotin complex (ABC) solution in PBS for 40 mins and then stained with DAB (IBA1: 4 mins and PV: 2 mins). For IBA1, the slides were dehydrated and coverslipped as previously described. For the PV they were counterstained with haematoxylin (#HEMM-35/21, Solmedia) for 20 seconds before dehydration and coverslipping.

SST

Tissue preparation was the same as described before, followed again by tissue blocking using 10% foetal bovine serum (FBS), 10% Normal Horse Serum (NHS), and 1% Bovine Serum Albumin (BSA) in 1% Triton-100-X (PBS-Tx) (w/v) for 2 hours. Slides were then incubated overnight at 4°Cwith the primary antibody SST (purified recombinant rabbit monoclonal, rabbit IgG, #ZRB1042, EMD Millipore Corporation, 1:100) in 2%FBS/2%NGS or 2% NHS/0.2%BSA + 1% Triton-100-X (PBS-Tx) (w/v).

On the following day slides were incubated with the ImmPRESS kit (#MP-7401, ImmPRESS horse anti-rabbit IgG polymer kit, peroxidase, 1-2 drops/brain) for 30 mins. The slides were then stained with DAB for 2 mins.

NFL Immunofluorescence (IF) staining

Sections were deparaffinised and rehydrated, then subjected to antigen retrieval. Following this, slides were blocked with 10% FBS/10%NGS/1%BSA + 1% Triton-100-X (PBS-Tx) (w/v) for 2 hours. The slides were then incubated with the primary antibody NFL (polyclonal, rabbit IgG, #171-002, 1:10,000) overnight at 4 degrees. After overnight incubation the sections were treated with the secondary antibody (Goat anti-rabbit, #A21245, Invitrogen Alexa Flora 647, 1:200) in 2%FBS/2%NGS or 2% NHS/0.2%BSA 1% + 1% Triton-100-X (PBS-Tx) (w/v) and protected from light for 90 mins. Following incubation, the slides were incubated with 4′,6-diamidino-2-phenylindole (DAPI) 1:1000 for 5 mins then and mounted with Prolong Diamond Antifade (Life technologies, #P36970) then sealed with all-purpose nail varnish and cured overnight at 4 degrees.

Immunostaining image analysis

Regions of interest (ROIs) were selected based on different cortical column orientations relative to the incident shockwave. The ROIs were primary auditory cortex (Au1), primary visual cortex (V1), retrosplenial cortex (RSC), and the CC (Fig. 1D). Density of NeuN positive neurons was assessed individually in each cortical layer. In all ROIs, a box was drawn to span the cortex from the bottom of layer 1 to the bottom of layer 6. Box width was set at 700 µm for the Au1, 400 µm for the RSC, and 500 µm for the V1 cortex. Boxes were divided into cortical layers and analysis was performed for both hemispheres. Each layer per region of interest was analysed separately. Neuronal density was calculated using a HALO® cytonuclear algorithm for the NeuN and the PV and SST were manually counted and presented as neuronal density cell/mm^2. For the NeuN analysis, the algorithm had a minimum positive stain threshold (a threshold colour), neuronal shape and size of a typical neuron was established using a control animal for each staining batch. This was to account for multi-day staining variation (e.g DAB staining can vary over days). This threshold was to discern the difference between positive neuron and the background brain parenchyma. These settings were then tested against other tissue sections of the that batch to assess suitability and accuracy. Once established these settings were applied to the rest of the brains for that batch.

For the NeuN analysis in the Au1 3 animals at 1-month post-injury were excluded due to tearing or staining artefacts in that region. For the V1 and RSC, 1 animal was not included at 7-days post-injury, and 3 animals at 1-month post-injury were excluded for the same reasons. The same regions of interest were used for GABAergic inhibitory interneurons analysis, but cells were counted manually. Results were presented as cellular density (cell/mm^2). For the Parvalbumin staining in the Au1, and the RSC, 1 animal was not included for the analysis due to staining artefacts in the tissue at 1-month post-injury. At 1-month post-injury 2 animals were excluded for the same reason in the V1. For astrocytic analysis, regions of interest had an overall box size comparable to those of the neuronal stains, e.g., 700 µm box width for Au1 cortex spanning from pia to white matter. The CC was outlined for analysis to be compared against the Naïve/Sham group. Using a HALO® algorithm for % area covered, a threshold between background and positive staining was established and used to calculate % area stained. For the RSC and V1, 2 animals were excluded from the 7-days post-injury group and 3 animals from the 1-month post-injury group due to tearing or staining artefacts. For the CC 1 animal was excluded from the 1-month post-injury group for similar reasons. Microglial density quantification was performed using a HALO microglial algorithm. For all regions, 2 animals were excluded from the 7-days and 1-month post-injury group due to staining artefacts. Microglial % area stained was calculated in a similar to that of the astrocytes. IBA1 % area stained had 4 animals excluded from the Naïve/Sham group due staining artefacts and 2 excluded from the 7-days post-injury group, for the same reasons. Neurofilament light (NFL)

was analysed in the corpus callosum using HALO's fluorescent intensity covered algorithm and intensity was normalised to two small low fluorescent areas of the periaqueductal grey (PAG). These served as the internal control. 1 animal was excluded from the 1-month and 3-months post-injury groups due to NFL staining artefacts. The data were presented as NFL intensity (% change from Naïve/Sham).

Surgery

Surgery was performed by the same experimenter to ensure consistency. Induction was performed using isoflurane (5% concentration, 2 L/min flow rate) inside an induction chamber, followed by stabilisation of respiratory rate using a face mask and titrating the concentration to a target of 80 breaths/minute. This was followed by an intraperitoneal injection of urethane (2.7 ml/kg, 1.35 g/ml, Sigma-Aldrich). Follow-up doses of 10% of the initial dose, were administered as necessary to ensure loss of eye-blink and paw-withdrawal reflexes. Atropine (0.66 ml/kg, 1% w/v) was administered subcutaneously to reduce mucous secretions.

After a minimum 1hour period, the scalp of the animal was shaved, and bupivacaine (7.5 mg/kg, Marcaine) was administered subcutaneously to the area for local analgesia. An incision was made to expose the skull, and tissue was cleared from the skull using blunt dissection. Throughout the anaesthetic induction and surgery, blood oxygen saturation and respiratory rate was monitored and normothermia was achieved using a physiological monitoring platform with closed-loop temperature control (Harvard Apparatus). Then throughout the course of the experiment, temperature was controlled using isothermal pads (Fisher Scientific) to prevent the introduction of electrical noise to the electrophysiology. Anaesthetic depth was frequently monitored by means of testing eye-blink and paw-withdrawal reflexes.

Recording Setup

The multielectrode EEG array (Neuronexus) used 500 µm diameter Platinum electrodes on a 15 µm thick Polyimide substrate, spanning 11.7 mm on the rostro-caudal and 9.6 mm on the left-to-right axis, with a minimum inter-electrode distance of 1.2 mm. Electrode impedance was tested to be below 10 kOhm at 1 kHz before recordings were made (this includes re-testing when the EEG electrode array was replaced in the subacute cohort between baselin and post-blast recordings).

Signals were digitized proximally to the animals' heads using a Neuronexus SmartLink^{TM} headstages. The reference and ground signals were obtained from copper wires of the headstage fitted through and wound around surgical needles inserted into the nuchal musculature, in a monopolar montage (referential recording). All signals were analog filtered between 1.1 Hz and 15 kHz and subsequently sampled at 30 kHz using a Neuronexus Smartbox Pro^{TM} acquisition board (16-bit A/D converter) interfacing with the Radiens Allego^{TM} acquisition software (Neuronexus) running on a Windows computer and saved to disk.

Example raw signals obtained with the EEG array for an animal in the Blast group are shown in Supplementary figure 6.

Electrophysiological Data Preprocessing

Data were preprocessed by the same investigator to ensure consistency. Rejection of line noise with the CleanLine method [6] was performed in two steps with different parameter values for the sliding window – 4 s windows with 50% overlap and 25 s windows with no overlap – that we empirically found resulted in satisfactory suppression of line noise. All other parameters were set at their default values. For the Artifact Subspace Reconstruction algorithm, we used a value of 12 for the burst criterion and 0.6 for the correlation criterion, with all other parameters set at default values (EEGLAB function *clean_artifacts*). Specifically, channels were flagged for rejection when the correlation with their estimate based on neighbouring channels fell below 0.6 for more than half the recording time, if they had more than 4 standard deviations of line noise relative to its signal based on the total channel population, and if they exhibited a flat signal for more than 5 seconds. Segments of signal were excluded from further analysis when their standard deviation exceeded 12 times that of calibration data (burst criterion). This criterion aims to remove large (outlier) activations that may be owed to motion or muscle artifacts, while the value used is within the range recommended in the literature (Chang et al. 2018) and is reasonable trade-off between rejecting artifacts and retaining sufficient data. Finally, segments were discarded if more than 25% of channels having power higher than 7 standard deviations relative to a robust power distribution. The segments identified for rejection were further confirmed visually within the EEGLAB graphical user interface. Datasets were excluded from further analysis in cases where line noise was not effectively suppressed, or the automated artifact rejection procedure led to more than 20% of the datapoints being rejected. 1 animal in the blast 1-month group and 2 animals in the sham 3-months group were excluded based on these criteria.

Statistical Analysis

For the electrophysiological results, statistical analysis was performed using Matlab™. For the acute electrophysiological cohort's global power and connectivity comparisons, we performed a permutation based paired *t*-test using functions from the Matlab™ Statistics and Machine Learning Toolbox (function *ttest*). For the chronic electrophysiological cohort's power comparisons at both the global and electrode/electrode pair level and for comparisons of global connectivity, a permutation based 2-way ANOVA (factor 1: injury group (blast vs sham), factor 2: timepoint (1-month post-blast vs 3-months post-blast, plus interaction term) test was performed [7], where *P*-values were calculated by comparing the observed F-values to the 95th percentile of a distribution of statistics obtained under a permutation of group labels (10,000 permutations). In the case of a significant interaction, post-hoc permutation-based independent samples Welch's unequal variances t-test were performed by adapting functions from the Matlab™ Statistics and Machine Learning Toolbox (function *ttest2*) between the pairs of interest (Blast vs Sham at 1-month, Blast vs Sham at 3-months and Blast at 1-month post-injury vs Blast at 3-months post-injury). To correct for multiple comparisons, the Bonferroni method was used for measures of global power and connectivity (n = 6 frequency bands), electrode-specific power for each band (n = 32 electrodes) and pairwise *t*-tests (n = 3 pairs). To assess differences at the level of individual edges, the timepoint factor was collapsed and an independent samples *t*-test was performed using the network-based-statistic toolbox [8]. Correction for multiple comparisons was performed using the toolbox implementation of the False Discovery Rate method with the corrected *P*-threshold set at P = 0.05 and 50000 permutations.

The histological data was analysed in GraphPad Prism (9.4.1). The control group consisted of naïve and sham pooled data as there were no significant differences between them. NeuN, PV, and SST cell densities were analysed per cortical layer for each region of interest. For these analyses, a Shapiro-Wilk normality test was performed followed by a one-way ANOVA (factor: time (Naïve/Sham, 6-hours, 7-days, 1-month post-blast vs 3-months post-blast)). Multiple-comparison tests were conducted for each region's layers using a Two-stage linear step-up procedure of Benjamini, Krieger and Yekutieli (BKY) False Discovery Rate (FDR) approach with $Q = 5\%$. Post-hoc comparisons (Uncorrected Fisher's Least Significant Difference (LSD)) were performed against the Naïve/Sham cohort. To investigate glial changes, the analysis was performed for each region of interest. A Shapiro-Wilk normality test was performed followed by a one-way ANOVA. To investigate glial activation due to blast in the CC a Shapiro-Wilk normality test and a Kruskal-Wallis test was performed. Neurofilament light data underwent normality tests followed by a one-way ANOVA. For each histological analysis, the data underwent an outlier identification check using the ROUT method provided by Prism (Q=1%). If a value was identified as an outlier, it was excluded from the dataset. Data were presented as ± SEM unless stated otherwise, and the threshold for significance was set at $P = 0.05$.

Investigators were not blinded to the outcomes assessments. No sample size or effect size calculations were performed.

Figures and Tables

**Blast Parameters Used**	
**Compression gas**	Compressed Air
**Mylar® burst membranes (µm)**	250
**Shock tube inner diameter (cm)**	5.9
**Burst pressure of Mylar® (kPa)**	1753.98 ± 9.16
**Sensor 2 incident peak pressure (kPa)**	232.72 ± 0.91
**Sensor 2 impulse (kPa ms)**	109.96 ± 0.76
**Sensor 2 positive duration (ms)**	1.38 ± 0.01
**Shock wave speed (m/s)**	560.52 ± 0.84
**Sensor 3 total pressure[a] (kPa)**	310 ± 10.90
**Sensor 3 impulse (kPa ms)**	245 ± 15.99
**Sensor 3 positive duration (ms)**	2.6 ± 0.34
**Rat snout distance from shock tube (cm)**	4.5
**Rat protrusion distance from PVS thoracic protection tube (cm)**	5
**LORR mean for Sham animals (mins)**	16.72 ± 1.76
**LORR mean for Blast animals (mins)**	31.82 ± 2.25

Supplementary Table 1: Blast characteristics

[a] Total pressure is the combination of both incident and reflected pressure is presented as mean ± SEM from 4 trials obtained during testing what the animal would receive. The total pressure is from 4 cm from the shock tube exit. LORR stands for Loss of Righting reflex.

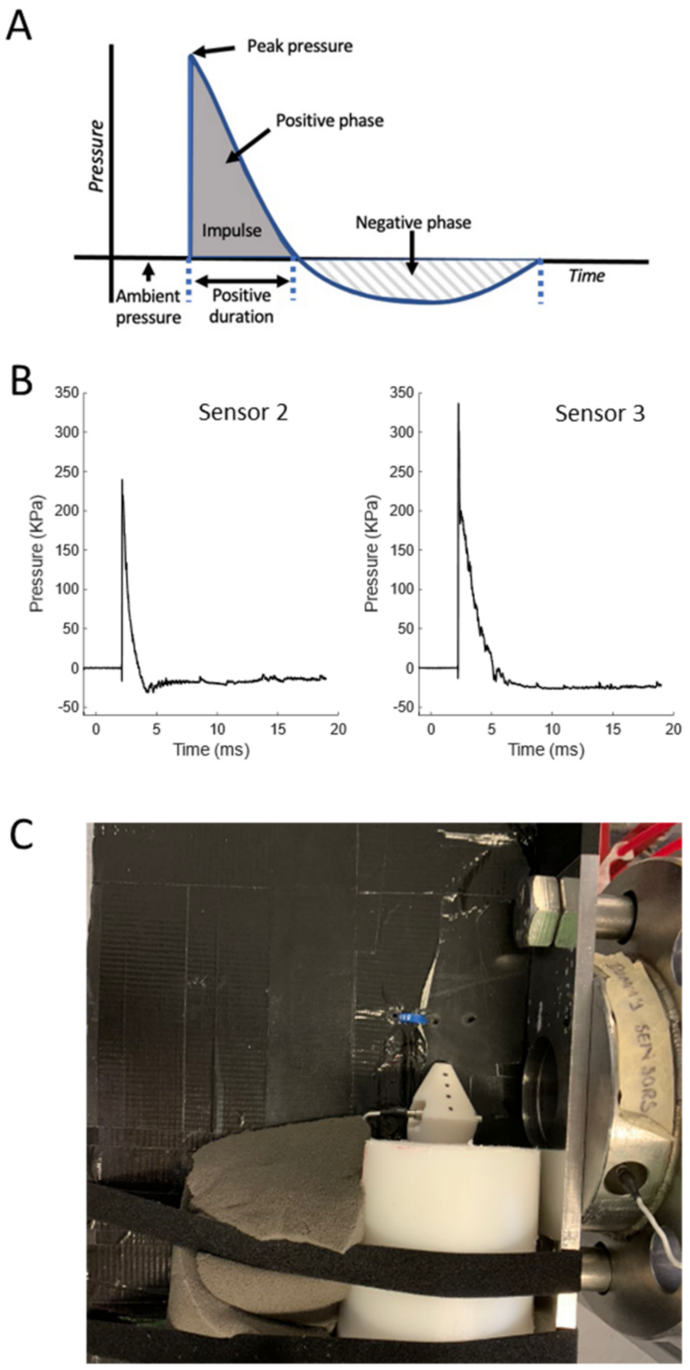

Supplementary figure 1 (A) Idealised Friedlander waveform. **(B)** Representative pressure-time graphs of the shock wave from, left graph; incident pressure sensor 2 and right graph; total pressure sensor 3. **(C)** 3rd Sensor 3D rat. 3D printed rat secured in place with hole screwed into the head for the sensor to sit flush with the edge of the "rat head" for accurate measurement of total pressure from the oncoming blast wave.

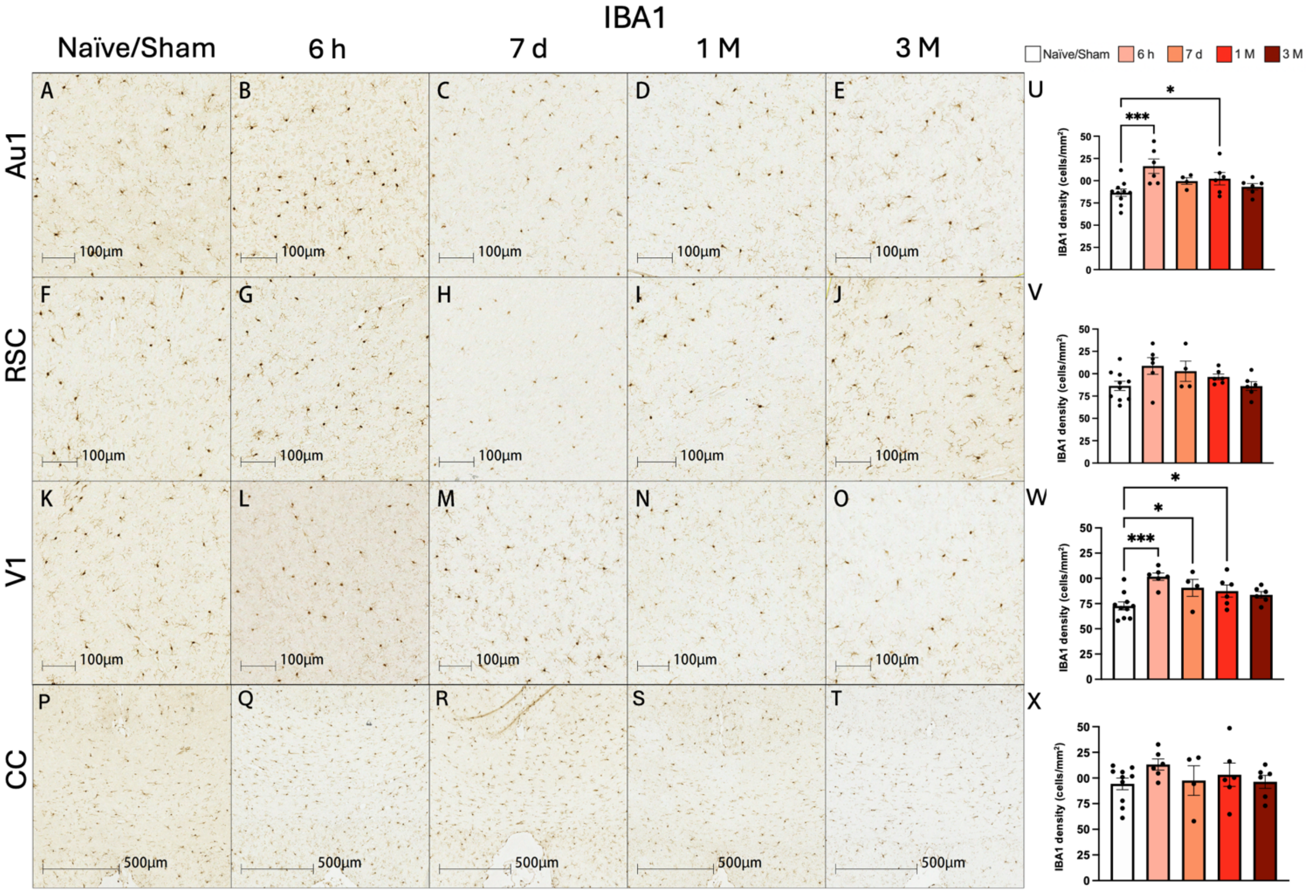

Supplementary figure 2 IBA1+ cell density increased in the Au1. IBA1+ cell density in all regions of interest over reported timepoints (A-E). IBA1+ staining of the auditory cortex. **(F-J)** IBA1+ staining in the retrosplenial cortex. **(K-O)** IBA1+ staining in the primary visual cortex (V1). **(P-T)** IBA1+ staining in the corpus callosum. **(A/F/K/P)** Naïve/Sham, **(B/G/L/M)** 6 hour post-injury, **(C/H/M/R)** 7-days post-injury, **(D/I/N/S)** 1-month post-injury, **(E/J/O/T)** 3-months post-injury. **(U-X)** quantification of IBA1+ for each region of interest using one-way ANOVA, (U) shows increased at 6 hours and 1 month post injury in the Au1. (Au1: Naïve/Sham: n = 13, 6 hour: n = 6, 7-day: n = 4, 1-month: n = 6, 3-months: n = 6. V1: Naïve/Sham: n = 13, 6 hour: n = 6, 7-day: n = 4, 1-month: n = 6, 3-months: n = 6. RSC: Naïve/Sham: n = 13, 6 hour: n = 6, 7-day: n = 4, 1-month: n = 6, 3-months: n = 6. CC: Naïve/Sham: n = 13, 6 hour: n = 6, 7-day: n = 4, 1-month: n = 6, 3-months: n = 6.

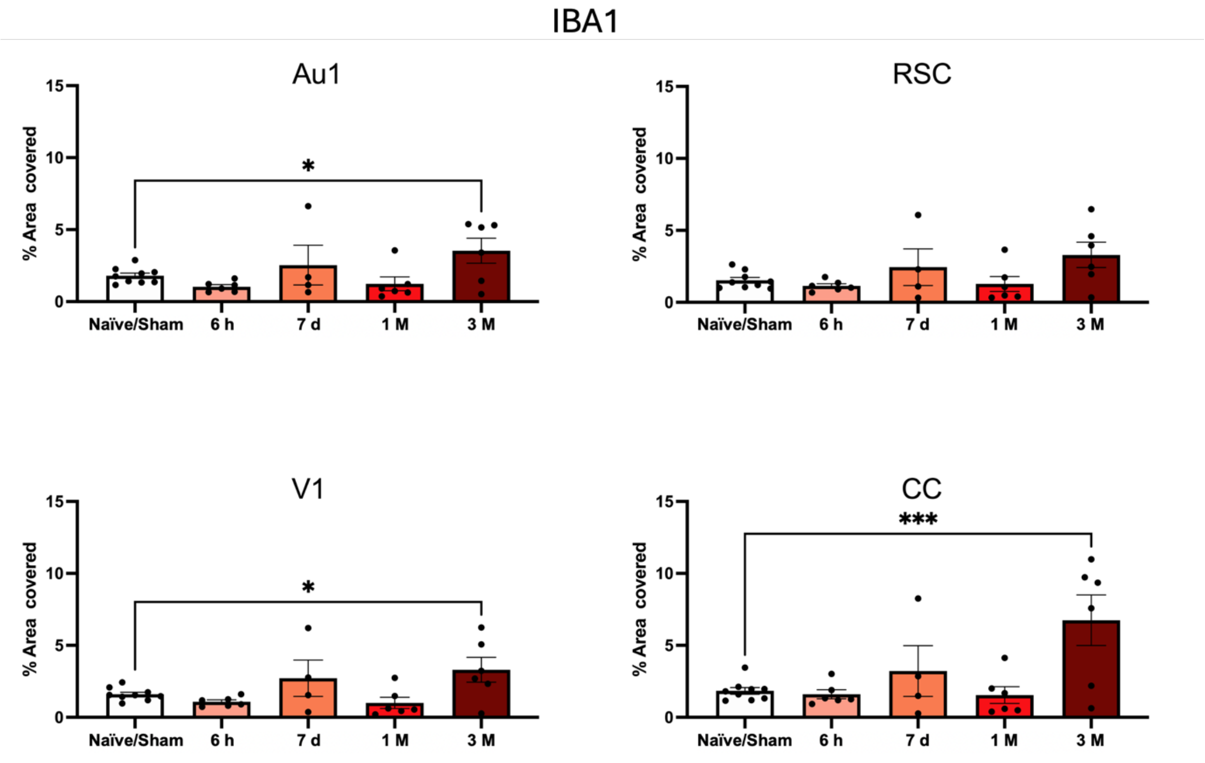

Supplementary figure 3 Analysis of IBA1 % area covered in all regions of interest at (6 hour, 7-days post-injury, 1-month and 3-months post-injury) in the primary auditory cortex (Au1), retrosplenial cortex (RSC), primary visual cortex (V1) and the corpus callosum (CC). One-way ANOVA revealed significant increase in % area stained at 3-months in the Au1, the V1 and the CC. Quantification of IBA1+ for each region of interest using one-way ANOVA data presented mean ± SEM, **P*<0.05 (Au1: Naïve/Sham: n = 9, 6 hour: n = 6, 7-day: n = 4, 1-month: n = 6, 3-months: n = 6. V1: Naïve/Sham: n = 9, 6 hour: n = 6, 7-day: n = 4, 1-month: n = 6, 3-months: n = 6. RSC: Naïve/Sham: n = 9, 6 hour: n = 6, 7-day: n = 4, 1-month: n = 6, 3-months: n = 6. CC: Naïve/Sham: n = 9, 6 hour: n = 6, 7-day: n = 4, 1-month: n = 6, 3-months: n = 6).

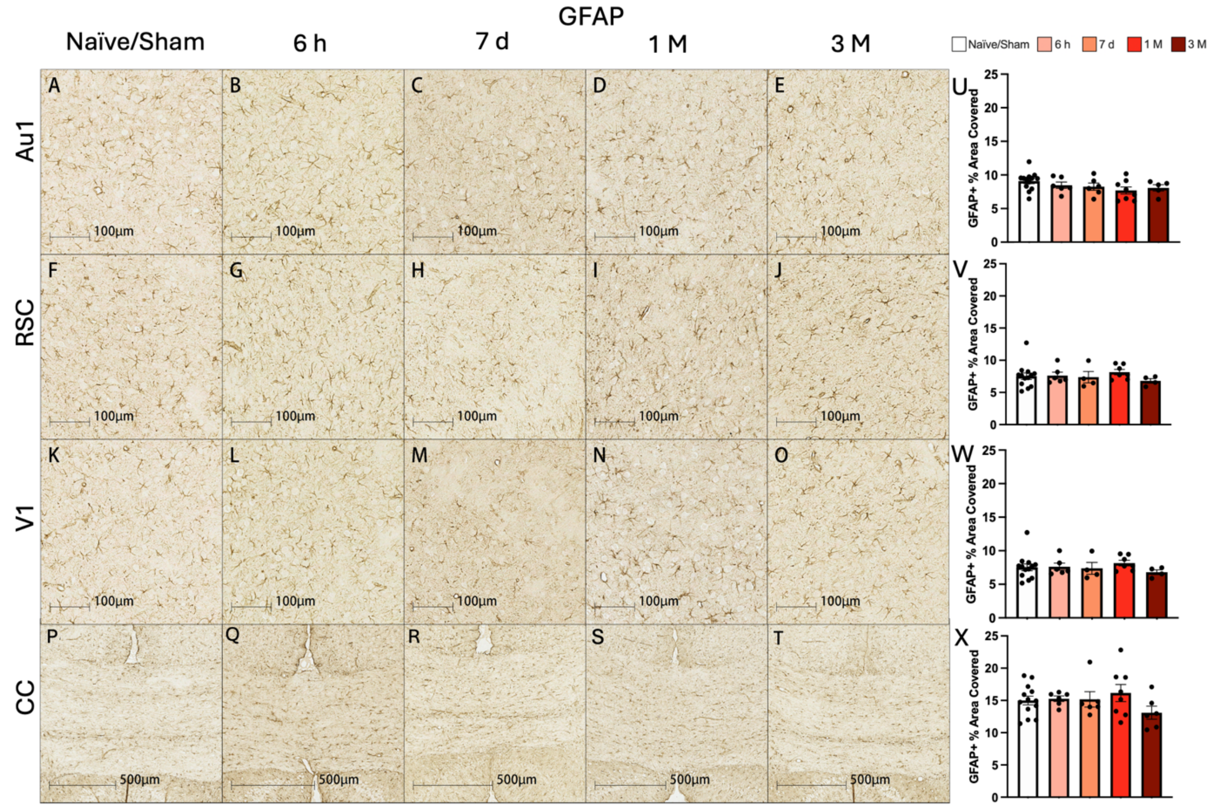

Supplementary figure 4 GFAP % area covered in all regions of interest (A-E) showed no changes. GFAP+ % staining in of the auditory cortex. **(F-J)**, retrosplenial cortex **(I-O)** GFAP staining in the primary visual cortex (V1) cortex. **(P-T)** GFAP staining in the corpus callosum (CC). **(A/F/K/P)** Naïve/Sham, **(B/G/L/M)** 6 hour post-injury, **(C/H/M/R)** 7-days post-injury, **(D/I/N/S)** 1-month post-injury, **(E/J/O/T)** 3-months post-injury. **(U-X)** quantification of IBA1+ for each region of interest using one-way ANOVA data presented mean ± SEM. *P<0.05, **P<0.01, ***P<0.001 and ****P<0.0001 (Au1: Naïve/Sham: n = 13, 6 hour: n = 6, 7-day: n = 6, 1-month: n = 8, 3-months: n = 6. V1: Naïve/Sham: n = 13, 6 hour: n = 6, 7-day: n = 4, 1-month: n = 5, 3-months: n = 6. RSC: Naïve/Sham: n = 13, 6 hour: n = 6, 7-day: n = 4, 1-month: n = 5, 3-months: n = 6. CC: Naïve/Sham: n = 13, 6 hour: n = 6, 7-day: n = 5, 1-month: n = 8, 3-months: n = 6)

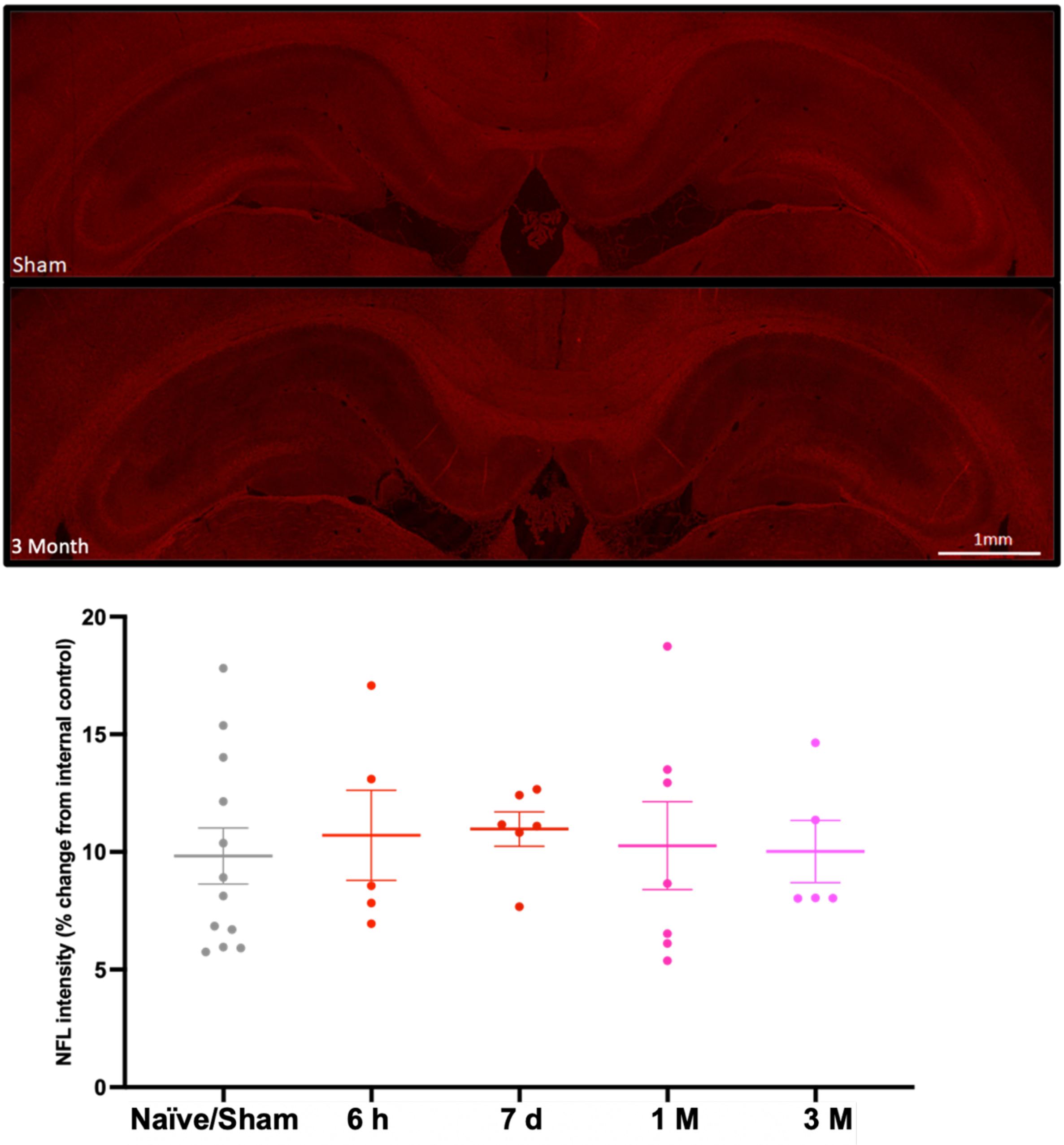

Supplementary figure 5 No neurofilament light fluorescence intensity changes in the corpus callosum over time. Representative staining of the corpus callosum of Alexa 546 of NFL of all time points post-injury is located above the quantification. Quantification of Alexa 546 immunofluorescence intensity for neurofilament staining in the whole of the corpus callosum showed no changes over time; quantified using one-way ANOVA. Data presented mean ± SEM. *$P<0.05$, **$P<0.01$, ***$P<0.001$ and ****$P<0.0001$ (Naïve/Sham: n = 13, 6 hour: n = 6, 7-day: n = 6, 1-month: n = 7, 3-months: n = 5).

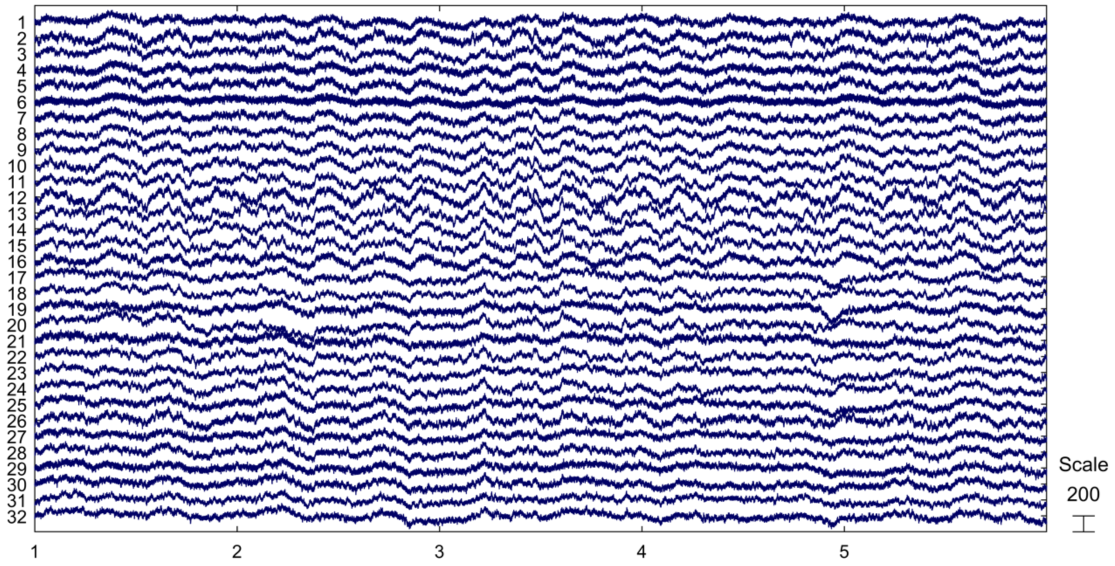

Supplementary figure 6 Example of raw EEG signals. Five seconds of data from all 32 channels are shown from an animal in the Blast group at 3 months. Data are sampled at 30 kHz and the vertical scale refers to 200 μV. The figure was made using the EEGLAB function *eegplot*.

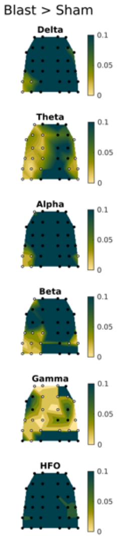

Supplementary figure 7 Sensitivity of electrode-level analysis of absolute power on channel interpolation. Topoplots depicting P-values for the main effect of group (permutation-based 2-way ANOVA, 10000 iterations), Bonferroni corrected for multiple comparisons (n = 32 channels) for all bands. Channels for an adjusted P-value below 0.05 are marked with white circles (blast 1-month: n = 13, blast 3-month: n = 20, sham 1-month n= 8, sham 3 -month n = 9). When rerunning the analysis without channel interpolation, results are robust and qualitatively highly similar to figure 3C of the main text.

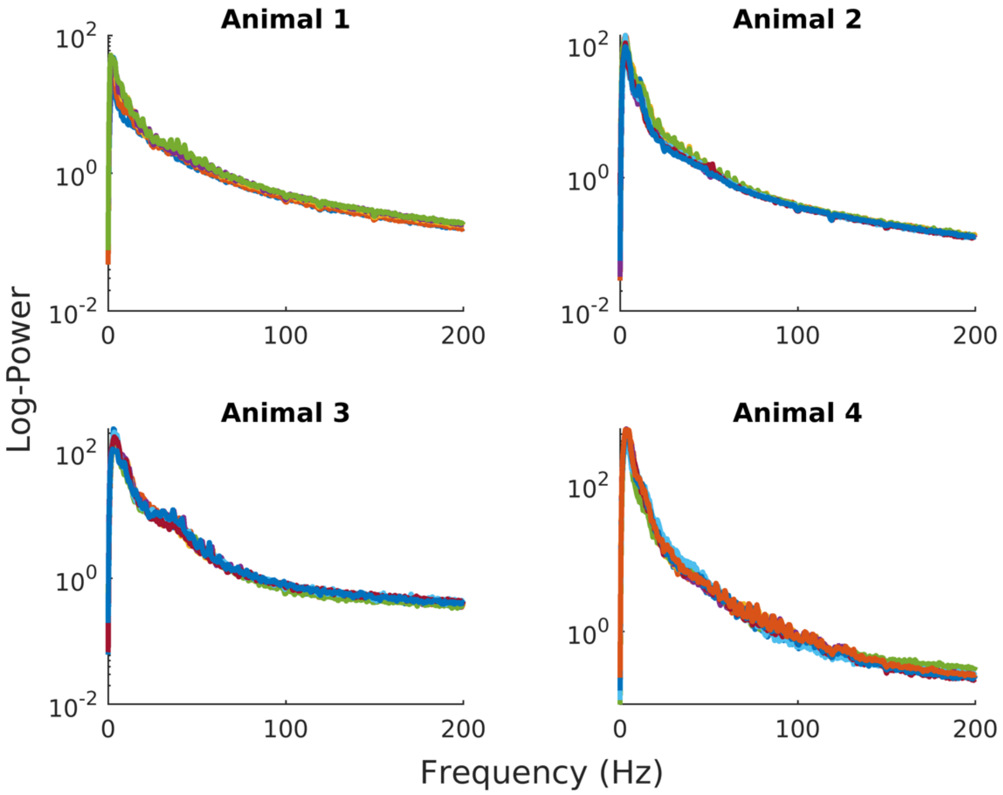

Supplementary figure 8 Robustness of signal over the length of the recording session. Plotted are mean power spectra across the electrode array for four representative subjects. Signals were robust across multiple runs during our recording sessions spanning approximately 6 hours. Different panels correspond to different subjects being recorded from during the same recording session, while different spectra within the same panel correspond to different runs from that recording session.

References:

1. Nguyen, T.-T., J.M. Wilgeroth, and W.G. Proud. *Controlling blast wave generation in a shock tube for biological applications*. in *Journal of Physics: Conference Series*. 2014. IOP Publishing.
2. Nguyen, T.-T., et al., *Experimental platforms to study blast injury.* Journal of the Royal Army Medical Corps, 2019. **165**(1): p. 33-37.
3. Nguyen, T.-T., et al., *Platform development for primary blast injury studies.* Trauma, 2018. **21**: p. 146040861877603.
4. Morton, D.B. and P.H. Griffiths, *Guidelines on the recognition of pain, distress and discomfort in experimental animals and an hypothesis for assessment.* Vet Rec, 1985. **116**(16): p. 431-6.
